# Mechanochemical modeling of exercise-induced skeletal muscle hypertrophy

**DOI:** 10.1371/journal.pcbi.1014691

**Published:** 2026-08-24

**Authors:** Ingvild S. Devold, Marie E. Rognes, Padmini Rangamani

**Affiliations:** 1 Department of Numerical Analysis and Scientific Computing, Simula Research Laboratory, Oslo, Norway; 2 K. G. Jebsen Centre for Brain Fluid Research, Oslo, Norway; 3 Department of Pharmacology, School of Medicine, University of California San Diego, La Jolla, California, United States of America; 4 Department of Mechanical and Aerospace Engineering, University of California San Diego, La Jolla, California, United States of America; Boston University, UNITED STATES OF AMERICA

## Abstract

Skeletal muscle displays remarkable plasticity, adapting its size and strength in response to mechanical loading, especially, from exercise. This process, known as hypertrophy, is fundamental to athletic training and rehabilitation, but is challenging to quantitatively predict due to its multifactorial, multiscale nature. Specifically, skeletal muscle hypertrophy results from an integration of macroscopic mechanical stimuli with the intracellular signaling pathways that govern muscle growth. In this work, we present a multiscale computational model that mechanistically integrates these mechanical and biochemical stimuli and offers a framework for predicting the outcomes of different types of exercise on skeletal muscle growth. The framework couples a transversely isotropic hyperelastic model for tissue-level mechanics with a system of ordinary differential equations representing the IGF1-AKT-mTOR-FOXO signaling pathway, a key regulator of protein synthesis and degradation. We link these scales using a volumetric growth model, where the signaling dynamics inform a growth tensor that drives changes in muscle cross-sectional area. This approach enables the simulation of long-term muscle adaptation, providing a mechanistic tool to investigate how different exercise protocols lead to macroscopic hypertrophy. Simulations from our model capture the temporal dynamics of hypertrophy under varying load protocols and highlight how feedback between protein synthesis and muscle growth regulates the dose-response relationship to prevent unbounded growth. Using muscle geometries derived from the Visible Human dataset, we study how human variations in muscle geometry affect hypertrophy. Finally, we demonstrate that the mechanochemical coupling between muscle geometry and signaling not only predicts macroscopic shape changes but also provides buffering from local signaling heterogeneity. Ultimately, this framework offers a predictive computational tool for optimizing training regimens and understanding the multiscale determinants of muscle adaptations.

## Author summary

Skeletal muscle naturally adapts its size and strength in response to physical activity, a process that is essential for both athletic training and rehabilitation. However, predicting how a specific training routine will alter a muscle’s shape remains difficult. This is because muscle growth relies on a complex, multiscale chain of events, where physical forces from movements trigger chemical signals that slowly rebuild the tissue over time. To better understand this process, we developed a computational model that links these underlying cellular signals directly to the macroscopic physical changes in the muscle. When simulating different exercise routines in realistic human muscle geometries, we found that the complex internal fiber architecture of the muscle causes it to grow unevenly. We also observed that the physical continuity of the tissue acts as a mechanical buffer, helping to coordinate noisy, localized signaling into smooth overall growth. Ultimately, this framework provides a foundational first step towards understanding how and where muscle adds mass, laying the groundwork for more advanced predictive tools.

## Introduction

**Fig 1:**
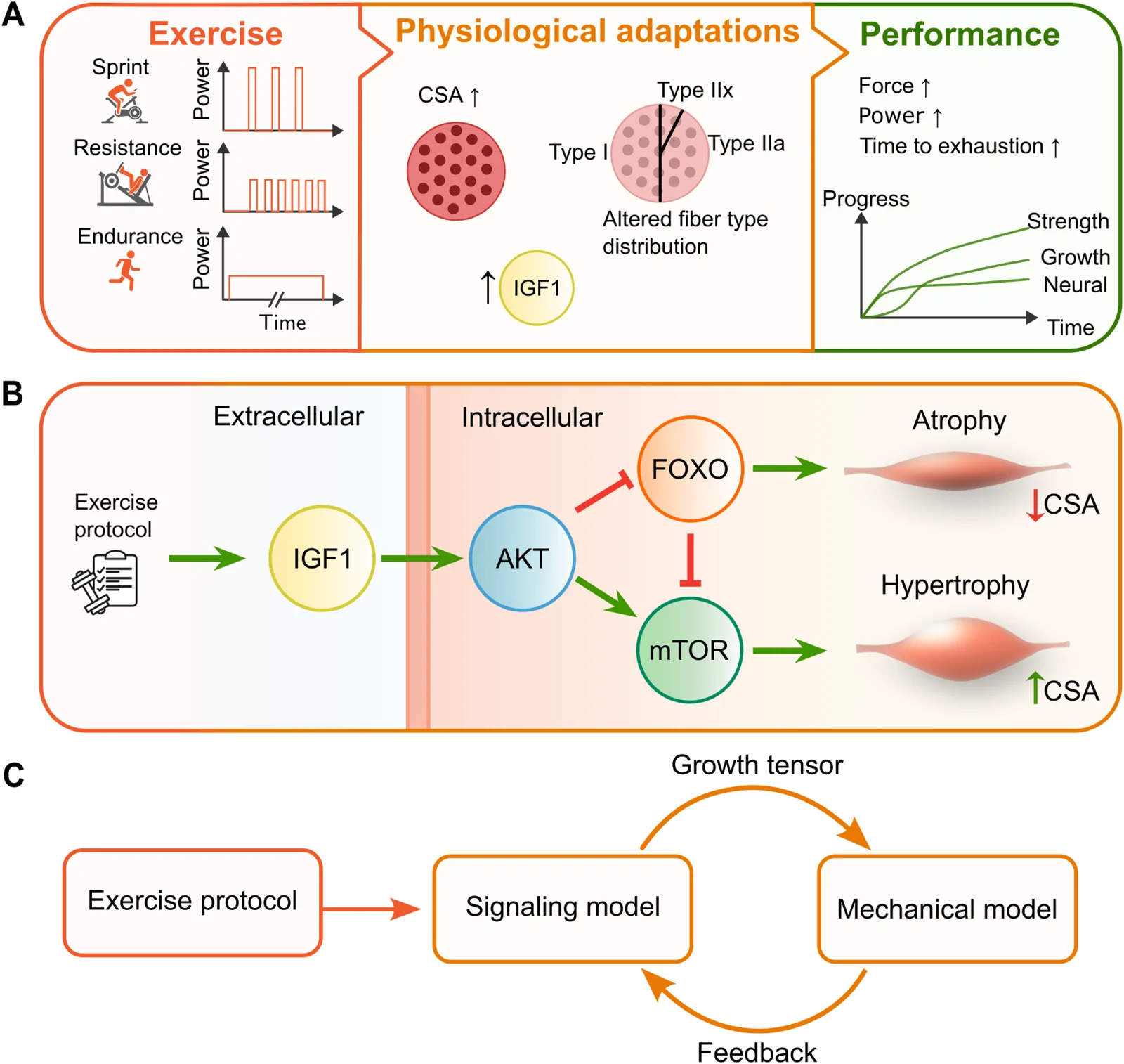
Conceptual overview of exercise-induced muscle adaptation and the modeling framework. **(A)** Different exercise types are characterized by distinct patterns of muscle activation, leading to physiological adaptations and ultimately affecting muscle performance. Here, “progress” refers to improvements in physical performance (strength), which is driven initially by rapid neural adaptations and later by structural muscle growth. **(B)** In this work, we focus on the IGF1-AKT signaling pathway, which is a key regulator of muscle hypertrophy. Mechanical stimuli through exercise activate IGF1, which in turn activates AKT. AKT promotes protein synthesis via mTOR and inhibits protein degradation via FOXO. **(C)** Schematic of the proposed multiscale model, coupling a signaling model with tissue-level mechanics to predict exercise-induced muscle growth. Abbreviations: CSA: cross-sectional area; IGF1: insulin-like growth factor 1; AKT: protein kinase B; mTOR: mechanistic target of rapamycin; FOXO: forkhead box transcription factor. *Figure partially created in BioRender.* Rangamani, P. (2026) [https://BioRender.com/wfzzhb7].

Skeletal muscle is remarkably plastic, constantly adapting its size and structure to mechanical stimuli resulting from different movements. This plasticity is not only critical for muscle’s ability to respond to the physical demands placed on it [1,2], but also critical to athletic training, rehabilitation, and overall health. Different types of exercise, such as high-intensity sprints, high-load resistance training, or long-duration endurance training, each induce distinct patterns of muscle activation that differ in duration, amplitude, and frequency (Fig 1A). Although the effects of exercise on skeletal muscle function are empirically well-documented [3], the underlying connections between molecular mechanisms and mechanical responses driving these adaptations remain poorly understood [4]. This is largely because they represent a profoundly multiscale phenomenon that requires an integration of tissue-level mechanics and subcellular biochemical signal transduction that has proven challenging to model.

The multiscale nature of muscle spans both spatial and temporal scales [4]. Spatially, muscle tissue is organized hierarchically, from the whole organ down to fascicles, individual muscle fibers, myofibrils, and finally to the sarcomere – the fundamental 2–3 µm contractile unit composed of actin and myosin filaments [5,6]. Temporally, muscle contraction is driven by rapid electrochemical events. This includes millisecond-timescale calcium transients triggering the ATP-fueled cross-bridge cycle, which in turn generates force and causes the relative sliding of the sarcomere filaments [7]. Over longer timescales, that span weeks to months, the cumulative effects of these contractions trigger intricate signaling pathways that regulate protein synthesis and degradation [8,9] and changes to metabolic pathways [10] ultimately remodeling the tissue structure. Mechanistically linking an exercise protocol to a long-term adaptation requires integrating our understanding of both muscle mechanics and cellular physiology.

From a mechanical perspective, foundational models of cellular-level force generation [11,12] have, with modern advances in medical imaging and computational resources, evolved into detailed three-dimensional models [13]. These models typically employ a hyperelastic material framework to accurately capture the large, non-linear deformations that muscle tissue undergoes during activity [14–16]. More recently, some models have begun to bridge scales by coupling cell-level models [17] with tissue-level mechanics [18–20]. From a physiological perspective, there are multiple signaling pathways at play in the muscle [8]. Among these, the IGF1-AKT-mTOR-FOXO signaling pathway has been identified as a central regulator of load-induced skeletal muscle hypertrophy [21–23]. In this cascade, mechanical loading through exercise stimulates the release of insulin-like growth factor (IGF1), which activates the protein kinase B (AKT) (Fig 1B). AKT, in turn, displays a dual response: it promotes protein synthesis via mechanistic target of rapamycin (mTOR) and inhibits protein degradation by suppressing the forkhead box transcription factor (FOXO), suggesting the presence of an incoherent feedforward loop [24,25]. The balance between these mTOR and FOXO-driven mechanisms dictates the net change in muscle protein and, consequently, muscle growth.

Computational models shed critical insights into how exercise can regulate muscle growth at different length and time scales. For short time-scale events, systems biology approaches have extensively mapped the calcium dynamics underlying excitation-contraction coupling [17,26], with recent work clarifying the role of store-operated Ca^2+^ entry (SOCE) in force modulation [7], the link between mechanical loading and metabolic sensors such as AMP-activated protein kinase (AMPK) [27], and the bidirectional coupling between exercise and mitochondrial dynamics [28]. However, such signaling models typically represent the muscle tissue as a homogeneous compartment, predicting hypertrophy as a scalar quantity without accounting for the complex spatial distribution of stress and strain within the 3D tissue.

Conversely, tissue-level mechanical models excel at resolving these local deformations using hyperelastic formulations. However, they predominantly operate under the assumption of mechanical equilibrium with fixed material reference states. By treating the tissue as a static material, these models neglect the dynamic, biochemical adaptation of the tissue constituents that occurs in response to mechanical loading over time. Bridging these domains requires overcoming a fundamental timescale mismatch: instantaneous mechanical stimuli trigger signaling events that accumulate over weeks to drive morphological change, which in turn alters the mechanical environment. The mathematical theory of volumetric growth [29–31] offers a rigorous kinematic framework to resolve this multiscale challenge. By decomposing the deformation gradient into elastic and growth contributions, this theory allows biologically driven mass accumulation from tissue growth to be coupled directly to continuum mechanics. The volumetric growth framework has previously found applications in several biological tissues [32,33], including cardiac [34,35] and brain tissue [36]. In the context of skeletal muscle, Villota-Narvaez et al. [37] found that a multiscale mechanobiological model, which integrates the IGF1-AKT signaling pathway with continuum mechanics via a growth tensor, accurately predicts muscle hypertrophy (CSA) results under various training conditions. However, their framework focused on idealized geometries and did not fully implement the cumulative growth kinematics required to robustly track residual stresses and shape evolution over long-term training protocols.

In this paper, we seek to address the following challenges: How can we simulate growth kinematics over timescales spanning several weeks to months while properly accounting for evolving residual stresses? How can we incorporate the effect of complex, non-uniform myofiber architectures found in realistic muscle anatomies and their influence on local growth? And finally, how can we leverage advances in computational mechanics to generate a predictive framework for muscle responses to different exercise schemes? To this end, we present a multiscale computational framework that couples tissue-level mechanics with the IGF1-AKT signaling pathway (Fig 1C). We ground our approach in the mathematical theory of volumetric growth [29,31], which allows biologically driven mass accumulation to be coupled directly to continuum mechanics. While our work builds on the mechanobiological model of Villota-Narvaez et al. [37], it extends the computational framework in two important directions. First, we adopt the cumulative growth framework of Goriely and Ben Amar [38], which ensures that each growth increment is applied to a stress-free configuration, providing a robust method for handling long-term adaptation. Second, we apply this coupled model to anatomically realistic muscle geometries derived from the Visible Human dataset [39–41], demonstrating the versatility of the approach. Using this framework, we demonstrate that high-intensity, high-frequency exercise protocols drive the most pronounced hypertrophy, reveal that complex muscle architectures result in non-uniform growth patterns, and that introducing heterogeneity in the signaling dynamics disrupts the overall growth. This integrated approach demonstrates how protocol-dependent signaling drives 3D tissue adaptation in realistic geometries, highlighting its predictive potential for both rehabilitation and peak performance sports.

## Methods

### Stress-strain relationships

Muscle tissue undergoes large deformations and exhibits a nonlinear stress-strain relationship under physiological loading. Its bundle-like structure results in a transversely isotropic material response, with distinct mechanical properties along and across the fiber directions ${𝐚}_{0}$ [42]. Accordingly, we model the mechanical behavior of muscle using a transversely isotropic hyperelastic framework [19]. In this framework, the stress response is derived from a strain energy density function, *W*, which depends on the tissue deformation. To define this relationship, we first introduce key kinematic quantities. Let $\Omega \subset {\mathbb{R}}^{3}$ denote a fixed reference domain (open, bounded) with coordinates 𝐗 and boundary ∂Ω. We let 𝐮=𝐱−𝐗 denote the displacement from this reference configuration to the current configuration. The deformation gradient is given by $𝐅=\nabla_{𝐗}𝐮+𝐈$, and the corresponding right Cauchy-Green deformation tensor is $𝐂={𝐅}^{T}𝐅$. The local volume change is measured by the Jacobian J=det(𝐅). The strain energy function for a transversely isotropic material is defined through the invariants of 𝐂: the first invariant $I_{1}=\text{tr}(𝐂)$ and the fourth pseudo-invariant $I_{4}={𝐚}_{0}\cdot 𝐂{𝐚}_{0}$. Finally, the fiber stretch is given by $\lambda =\sqrt{I_{4}}$.

The stress experienced by the material is quantified with respect to the reference configuration by the first and second Piola–Kirchhoff stress tensors, 𝐏 and 𝐒, which are derived from a strain energy function as $𝐏=\frac{\partial W}{\partial 𝐅}$ and $𝐒=2\frac{\partial W}{\partial 𝐂}$, respectively, and are related through the deformation gradient as 𝐏=𝐅𝐒. In the constitutive model presented by Röhrle et al. [19], the second Piola–Kirchhoff stress tensor is given by the sum of isotropic, anisotropic and active contributions:


$$\begin{array}{c} 𝐒=\underset{\equiv {𝐒}^{iso}}{\underbrace{c_{1}𝐈+c_{2}(I_{1}𝐈-𝐂)-pJ{𝐂}^{-1}}}+\underset{\equiv {𝐒}^{aniso}}{\underbrace{\left(\frac{\sigma_{passive}}{I_{4}}f_{passive}(I_{4})\right)(a_{0}⊗a_{0})}}+\underset{\equiv {𝐒}^{active}}{\underbrace{\alpha \left(\frac{\sigma_{active}}{I_{4}}f_{active}(I_{4})\right)(a_{0}⊗a_{0})}}. \end{array}$$ (1)

In (1), the isotropic term ${𝐒}^{iso}$ defines a Mooney–Rivlin material with material constants *c*_1_ and *c*_2_ (Table 1) and hydrostatic pressure *p*. The term ${𝐒}^{aniso}$ accounts for passive fiber anisotropy, while ${𝐒}^{active}$ represents active fiber contraction.

**Table 1:** Material constants and force-length relationship parameters for the mechanical muscle tissue model.

Parameter	Symbol	Value	Unit	Reference
Mooney–Rivlin material constants	*c*_1_	0.01	M Pa	[18]
	*c*_2_	0.01	M Pa	[18]
Maximum isometric stress	$\sigma_{passive}$	0.3	M Pa	[14,18]
	$\sigma_{active}$	0.3	M Pa	[14,18]
Coefficients $f_{passive}$	$\gamma_{1}$	0.05	–	[14]
	$\gamma_{2}$	6.6	–	[14]
Optimal fiber stretch	$\lambda_{opt}$	1.4	–	[14,18]
Spring stiffness	*k*	0.1	M Pa	–

**Fig 2:**
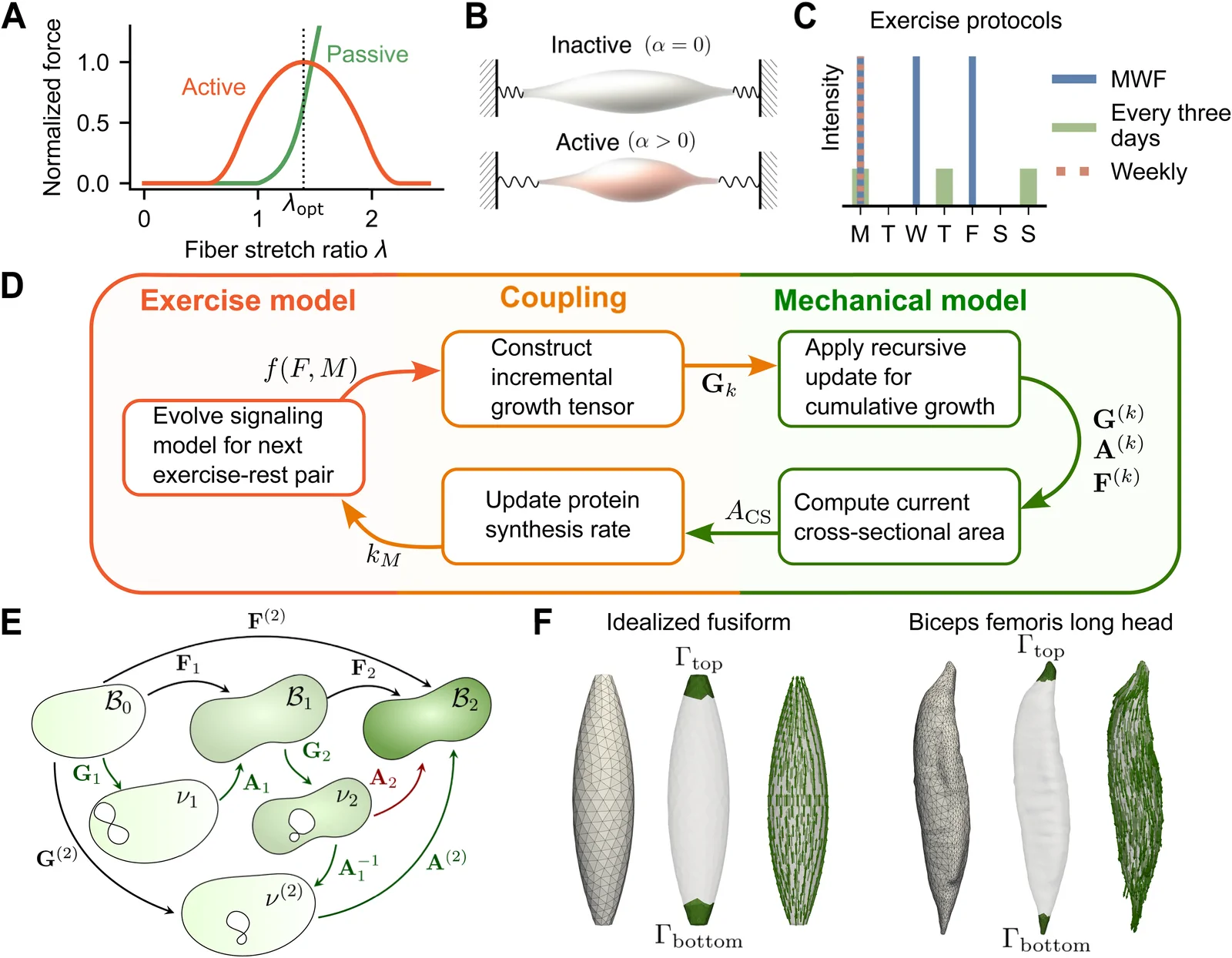
Computational model components and workflow. **(A)** The passive and active fiber forces are dependent on stretch λ. **(B)** A muscle contraction, showing the inactive (α=0) and active (α>0) states. **(C)** Illustration of one week of the three exercise protocols used in growth simulations. **(D)** The coupling scheme between signaling and growth mechanics used in our computational framework. The exercise model determines an incremental growth tensor ${𝐆}_{k}$, which updates the mechanical model through the cumulative growth framework. The resulting cross-sectional area $A_{CS}$ feeds back to modulate the signaling model via the protein synthesis rate $k_{M}$. **(E)** Schematic of the cumulative growth framework, based on the multiplicative decomposition of deformation, 𝐅=𝐀𝐆. Between growth steps, computational unloading (${𝐀}_{1}^{-1}$, green pathway) is required to avoid applying new deformation to a residually stressed state $\nu_{2}$ (red arrow). **(F)** Example geometries showing the computational mesh, surfaces for boundary conditions ($\Gamma_{\text{top}},\Gamma_{\text{bottom}}$), and prescribed fiber architecture.

The force-length relationships, *f*_passive_ and *f*_active_, depend on the fiber stretch λ and follow a standard normalized model [43] where the maximal active force is generated at an optimal fiber stretch, $\lambda_{\text{opt}}=1.4$ (Fig 2A) [14,18]. Specifically, we define the passive and active components by the following piecewise exponential and polynomial functions, respectively [14,18]:


$$\begin{array}{ccc} f_{passive}(\lambda ) & = & \left\{ \begin{array}{cc} 0 & \;\lambda ⩽1,\\ \gamma_{1}(e^{\gamma_{2}(\lambda -1)}-1) & \;1<\lambda <1.4,\\ \gamma_{3}\lambda +\gamma_{4} & \;\lambda ⩾1.4, \end{array}\right. \end{array}$$ (2a)

$$\begin{array}{ccc} f_{active}(\lambda ) & = & \left\{ \begin{array}{cc} 0 & \;\lambda ⩽0.4\lambda_{opt},\\ 9(\lambda /\lambda_{opt}-0.4{)}^{2} & \;0.4\lambda_{opt}<\lambda ⩽0.6\lambda_{opt},\\ 1-4(1-\lambda /\lambda_{opt}{)}^{2} & \;0.6\lambda_{opt}<\lambda ⩽1.4\lambda_{opt},\\ 9(\lambda /\lambda_{opt}-1.6{)}^{2} & \;1.4\lambda_{opt}<\lambda ⩽1.6\lambda_{opt},\\ 0 & \;\lambda >1.6\lambda_{opt}, \end{array}\right. \end{array}$$ (2b)

where the parameters $\gamma_{1}$ and $\gamma_{2}$ are given in Table 1, while $\gamma_{3}$ and $\gamma_{4}$ are chosen to ensure continuity and differentiability. Upon activation (α>0), the active stress component generates contraction along the fiber direction ${𝐚}_{0}$ (Fig 2B, 2F).

### Mechanical equilibrium equations

To determine the muscle tissue’s mechanical state for a given activation level α, we solve for the displacement-pressure pair (𝐮,p) satisfying the governing equations of static equilibrium defined relative to the reference domain Ω:


$$\begin{array}{cc} \nabla \cdot 𝐏 & =0\;\;\text{in }\Omega , \end{array}$$ (3a)

$$\begin{array}{cc} J-1 & =0\;\;\text{in }\Omega , \end{array}$$ (3b)

$$\begin{array}{cc} 𝐏\cdot 𝐍 & ={𝐓}_{R}\;\;\text{on }\partial \Omega_{R}=\Gamma_{top}\cup \Gamma_{bottom}, \end{array}$$ (3c)

$$\begin{array}{cc} 𝐏\cdot 𝐍 & =0\;\;\text{on }\partial \Omega ⧵\partial \Omega_{R}. \end{array}$$ (3d)

Here, (3a) represents mechanical equilibrium in the absence of body forces, (3b) enforces incompressibility, and 𝐍 is the outward unit normal to the boundary ∂Ω. The Robin boundary $\Omega_{R}$ includes the two ends of the muscle, denoted as $\Gamma_{top}$ and $\Gamma_{bottom}$, respectively. On these specific surfaces, we apply the nominal traction ${𝐓}_{R}$ via the Robin boundary condition (3c). We assume that the physical Cauchy traction 𝐭, which is defined relative to the deformed boundary surface with unit normal 𝐧, is negatively proportional to the displacement in the normal direction with proportionality constant k⩾0:


$$\begin{array}{c} 𝐭=-k(𝐧⊗𝐧)𝐮 \end{array}$$

This condition models the muscle connecting to elastic springs with stiffness *k* (Fig 2B), allowing contraction while stabilizing the nonlinear solver. We then define ${𝐓}_{R}$ as the pull-back of 𝐭 to the reference configuration:


$$\begin{array}{c} {𝐓}_{R}(𝐗)=\|J{𝐅}^{-T}𝐍(𝐗)\|𝐭(𝐱), \end{array}$$

where we have used Nanson’s formula to map between area in the reference and deformed configurations. The remaining boundaries are traction-free (3d).

### Signaling model

The IGF1-AKT-mTOR-FOXO signaling pathway is a central regulator of muscle mass, balancing protein synthesis and degradation [8]. To model the biochemical drivers of hypertrophy, we adopt the signaling model from Villota-Narvaez et al [44], which in turn builds upon a simplified representation of the pathway by Schiaffino and Mammucari [22] (Fig 1B).

The model consists of a system of ordinary differential equations (ODEs) describing the pathway’s key components. Let *I*, *A*, *F*, and *M* denote the normalized populations of IGF1, AKT, FOXO, and mTOR, respectively. Their dynamics are modeled using a Lotka–Volterra framework that captures the activating and inhibiting interactions between them through intrinsic growth rates ($a_{i}$), self-inhibition rates ($b_{i}$), and coupling strengths ($c_{ij}$) (Table 2):


$$\begin{array}{ccc} \frac{dI}{dt} & = & I(a_{I}(t-t_{delay})-b_{I}I), \end{array}$$ (4a)

$$\begin{array}{ccc} \frac{dA}{dt} & = & A(a_{A}(t)-b_{A}A+c_{AI}I), \end{array}$$ (4b)

$$\begin{array}{ccc} \frac{dF}{dt} & = & F(a_{F}-b_{F}F-c_{FA}A), \end{array}$$ (4c)

$$\begin{array}{ccc} \frac{dM}{dt} & = & M(a_{M}-b_{M}M+c_{MA}A-c_{MF}F). \end{array}$$ (4d)

**Table 2:** Baseline parameter values for the IGF1-AKT-mTOR-FOXO signaling model, adapted from [44], with $k_{M}^{0}$ calibrated, and initial conditions adjusted to equilibrium values.

Parameter	Symbol	Value	Unit
Intrinsic growth rate	$a_{I}^{0}$	$9.000\times 10^{-2}$	$h^{-1}$
	$a_{A}^{0}$	$4.875\times 10^{-1}$	$h^{-1}$
	$a_{F}$	$1.068\times 10^{-2}$	$h^{-1}$
	$a_{M}$	$4.635\times 10^{-3}$	$h^{-1}$
Self-inhibition rate	$b_{I}$	2.0	$h^{-1}$
	$b_{A}$	$5.000\times 10^{-1}$	$h^{-1}$
	$b_{F}$	$2.000\times 10^{-2}$	$h^{-1}$
	$b_{M}$	$1.000\times 10^{-2}$	$h^{-1}$
Coupling strength	$c_{AI}$	$2.846\times 10^{-1}$	$h^{-1}$
	$c_{FA}$	$2.000\times 10^{-3}$	$h^{-1}$
	$c_{MA}$	$1.139\times 10^{-3}$	$h^{-1}$
	$c_{MF}$	$2.500\times 10^{-3}$	$h^{-1}$
Min. myofibril population	$N_{\min}$	0.5	–
Max. myofibril population	$N_{\max}$	1.3	–
Threshold value FOXO	$F^{*}$	0.434	–
Threshold value mTOR	$M^{*}$	0.469	–
Protein synthesis rate	$k_{M}^{0}$	$1.000\times 10^{-2}$	$h^{-1}$
Protein degradation rate	$k_{F}$	$1.900\times 10^{-2}$	$h^{-1}$
$a_{I}(t)$ scale parameter	η	0.04	$(h\cdot kgf{)}^{-1}$
$a_{I}(t)$ max. muscle force	$F_{max}$	150	kgf
$a_{A}(t)$ scale parameter	$\tau_{s}$	6	h
$a_{A}(t)$ delay parameter	$\tau_{d}$	17	h
Initial condition IGF1	*I*_0_	$4.500\times 10^{-2}$	–
Initial condition AKT	*A*_0_	1.001	–
Initial condition FOXO	*F*_0_	$4.339\times 10^{-1}$	–
Initial condition mTOR	*M*_0_	$4.690\times 10^{-1}$	–
Initial condition myofibrils	*N*_0_	1.001	–

The rate of change of the myofibril population *N* reflects a balance between mTOR-driven synthesis and FOXO-driven degradation. We first define the unconstrained signaling rate, *f*(*F*, *M*), which captures these contributions when the signals exceed their respective thresholds, $M^{*}$ and $F^{*}$:


f(F,M)={−0if F<F* and M<M*,−kM(M−M*)−kF(F−F*)if F⩾F* and M⩾M*,−kF(F−F*)if F⩾F* and M<M*,−kM(M−M*)if F<F* and M⩾M*. (5)

To ensure the myofibril population remains within an admissible range $\left[N_{\min},N_{\max}\right]$, we further impose:


$$\begin{array}{c} \frac{dN}{dt}=\left\{ \begin{array}{cc} 0 & \text{if }N⩽N_{\min}\text{ and }f(F,M)<0\text{ or }N⩾N_{\max}\text{ and }f(F,M)>0,\\ f(F,M) & \text{otherwise}. \end{array}\right. \end{array}$$ (6)

This balance is driven by the protein synthesis rate $k_{M}$ (Fig C in S1 Appendix) and degradation rate $k_{F}$, with contributions effective only when mTOR and FOXO exceed their respective thresholds, $M^{*}$ and $F^{*}$.

Exercise is modeled as a stimulus that modulates the intrinsic growth rates of both IGF1 and AKT. First, the IGF1 growth rate, $a_{I}(t)$, is elevated during exercise according to the relation


$$\begin{array}{c} a_{I}(t)=\left\{ \begin{array}{cc} \eta I_{ex}F_{\max} & \text{ if }t\in \text{ exercise session},\\ a_{I}^{0} & \text{ otherwise}, \end{array}\right. \end{array}$$ (7)

where η is a scaling factor, $I_{ex}\in [0,1]$ is the exercise intensity factor, $F_{\max}$ is the muscle’s maximum force capacity, and $a_{I}^{0}$ denotes the baseline rate (Table 2). The stimulus is subject to a physiological delay, which we represent by $t_{delay}$ in the governing equation for IGF1 (4a) and set to $t_{delay}=12$h. Second, the intrinsic growth rate of AKT, $a_{A}(t)$, responds to exercise with a transient increase defined by


$$\begin{array}{c} a_{A}(t)=a_{A}^{0}+\max\left\{0,\;\frac{1}{2}\int_{t_{s}}^{t}\left(\frac{-1}{\tau_{s}}-\frac{\tau -t_{s}-\tau_{d}}{\tau_{s}^{2}}\right)\exp\left[\frac{\tau -t_{s}-\tau_{d}}{\tau_{s}}\right]\;d\tau \right\}. \end{array}$$ (8)

In this expression, $\tau_{d}$ is a delay parameter, $\tau_{s}$ a time-scaling factor, and $t_{s}$ marks the start time of the most recent exercise session. This formulation creates a transient increase in AKT’s growth rate that decays over several hours, while the max operation ensures $a_{A}(t)$ does not fall below its baseline level, $a_{A}^{0}$.

### Exercise protocols

We simulated three distinct exercise regimens to test protocol-dependent adaptation (Fig 2C). These were: a high-intensity, high-frequency protocol (1h at 80% intensity, 3x/week, MWF) based on DeFreitas et al. [45]; a high-intensity, low-frequency protocol (1h at 80% intensity, 1x/week); and a low-intensity, medium-frequency protocol (4h at 20% intensity, every three days). The intensity factor $I_{ex}$ was set to 0.8 and 0.2 for the high-intensity and low-intensity regimes, respectively. For all protocols, there is an initial week without exercise to establish baseline conditions, followed by eight weeks of the specified exercise regimen.

### Initial conditions

The initial conditions ($I_{0},A_{0},F_{0},M_{0},N_{0}$) for the signaling model represent a homeostatic, pre-exercise steady state. To determine these values, we simulated the ODE system (4) with baseline parameter values and no external stimulus ($a_{I}(t)=a_{I}^{0},a_{A}(t)=a_{A}^{0}$) until a stable equilibrium was reached. These equilibrium values, listed in Table 2, were used for all subsequent simulations.

### Spatial variations in signaling

To capture cell-to-cell variability and represent the muscle as a heterogeneous tissue, we introduce spatial variation in the signaling model. Specifically, spatial heterogeneity and inter-individual variability are emulated by perturbing selected signaling parameters locally. Each baseline parameter *p*_0_ (Table 2) is replaced by a spatially varying parameter


$$\begin{array}{c} p(𝐱)=p_{0}(1+\delta (𝐱)), \end{array}$$

where δ(𝐱) is a random field sampled from a uniform distribution on [−Δ,Δ] (e.g., Δ=0.1 for ±10% variation). Perturbations were drawn independently per mesh cell to represent within-muscle heterogeneity, and sampled once per simulation to simulate between-subject variability.

### Sensitivity analysis

We performed a Sobol sensitivity analysis [46] using the SALib Python library [47,48] to identify the most influential parameters in the signaling model. The final myofibril population (*N*) after a 15-day simulated exercise protocol was selected as the primary output metric, serving as a computationally efficient proxy for muscle growth. For the analysis, each parameter was sampled from a uniform distribution spanning 50% to 200% of its baseline value. To ensure a robust assessment, the analysis included first- and second-order effects with 10,000 samples, resulting in 300,000 total model evaluations.

### Model coupling

To capture the interplay between molecular signaling and tissue mechanics during skeletal muscle hypertrophy, we couple the subcellular signaling pathway with the macroscopic mechanical model. It is important to note that in the current framework, growth is driven directly by the signaling output of the ODE model rather than by local mechanical stimuli such as computed tissue stress or strain. Consequently, aggregate phenomenological parameters governing the whole-muscle exercise stimulus, such as the maximum force capacity $F_{\max}$, are decoupled from the maximum isometric stress parameter $\sigma_{active}$ that strictly dictates tissue-level continuum mechanics. We implement this specific form of bidirectional coupling through two primary mechanisms: (1) the signaling dynamics inform a volumetric growth tensor that drives changes in muscle geometry, and (2) the resulting macroscopic change in muscle size provides feedback to the signaling pathway by modulating a key parameter – the protein synthesis rate $k_{M}$ (Fig 2D). These two coupling mechanisms are detailed in the following sections.

### From signaling to macroscopic growth through the growth tensor

Molecular signals are translated into macroscopic growth by linking the myofibril population *N*(*t*) from the signaling model to the tissue’s deformation. This coupling is formalized using the theory of volumetric growth, where the total deformation gradient 𝐅 is decomposed into a growth part 𝐆 and an elastic part 𝐀, such that 𝐅=𝐀𝐆 [29,31]. In this decomposition, the growth tensor 𝐆 represents the local (potentially incompatible) addition of mass driven by biology, while 𝐀 is the subsequent elastic deformation required to maintain mechanical equilibrium and ensure the tissue remains a compatible, continuous body. Importantly, the biological tissue is assumed incompressible under elastic loading (3b). Therefore, the incompressibility constraint is imposed strictly on the elastic tensor, $J_{e}=\det(𝐀)=1$. This allows the total tissue volume J=det(𝐅) to increase dynamically in response to exercise-driven growth.

To determine 𝐆, we first calculate a scalar growth multiplier, *G*(*t*). The core assumption linking *N*(*t*) to *t*he continuum grow*t*h is that the myofibril population is directly proportional to the muscle cross-sectional area (CSA), $A_{CS}(t)$, via a constant κ, such that $N(t)=\kappa A_{CS}(t)$. The explicit formulation for calculating $A_{CS}(t)$ in the deformed geometry is developed later in the text (13). From the change in myofibrils ΔN=f(F,M)Δt predicted by the signaling model over a time step Δt, the growth multiplier is defined as:


$$\begin{array}{c} G(t)=1+\frac{\Delta N}{N(t-\Delta t)}=1+\frac{f(F,M)\Delta t}{\kappa A_{CS}(t-\Delta t)}, \end{array}$$ (9)

where N(t−Δt) is the myofibril population at the previous time step. This scalar multiplier then defines the anisotropic growth tensor 𝐆:


$$\begin{array}{c} 𝐆(t)=\sqrt{G(t)}𝐈+(1-\sqrt{G(t)}){𝐚}_{0}⊗{𝐚}_{0}. \end{array}$$ (10)

This formulation ensures that growth occurs exclusively in the plane perpendicular to the fiber direction ${𝐚}_{0}$, consistent with an increase in CSA [32]. Crucially, the 3D continuum framework translates the zero-dimensional scalar output of the signaling model into physical, non-uniform tissue deformations determined by local fiber architecture.

### Feedback from mechanics to signaling

The bidirectional coupling is completed by a feedback mechanism where the mechanical state of the muscle modulates the signaling pathway. Concretely, the protein synthesis rate $k_{M}$ is set to depend on the current CSA of the muscle $A_{CS}$. We define the normalized CSA as $A_{CS}^{*}(t)=A_{CS}(t)/A_{CS}(0)$, where $A_{CS}(0)$ is the initial area. The synthesis rate is then updated according to a Hill-type inhibitory function:


$$\begin{array}{c} k_{M}(t)=2k_{M}^{0}\left(1-\frac{(A_{CS}^{*}{)}^{n}}{K_{A}^{n}+(A_{CS}^{*}{)}^{n}}\right). \end{array}$$ (11)

With a Hill coefficient of *n* = 4 and a half-max constant of $K_{A}=1$, this function establishes homeostatic control. The synthesis rate returns to its baseline value when $A_{CS}^{*}=1$, decreases during hypertrophy ($A_{CS}^{*}>1$) to limit excessive growth, and increases during atrophy ($A_{CS}^{*}<1$) to stimulate growth back towards the baseline. This bounded, Hill-type regulation provides a simple, yet physiologically plausible feedback loop for muscle size adaptation (Figs D,E in S1 Appendix).

### Implementation of cumulative growth

To model the long-term effects of incrementally applied growth, we adopt the cumulative growth framework of Goriely and Ben Amar [38]. A key requirement of this framework is that each new growth increment, ${𝐆}_{k}$, must be applied to a stress-free configuration. Because the tissue is generally under residual stress from previous growth steps, this requires computationally “unloading” the accumulated elastic deformation before applying the next increment (Fig 2E). After *k* growth steps, the total deformation ${𝐅}^{\left(k\right)}$ is decomposed into a total elastic tensor ${𝐀}^{\left(k\right)}$ and a total growth tensor ${𝐆}^{\left(k\right)}$, such that ${𝐅}^{\left(k\right)}={𝐀}^{\left(k\right)}{𝐆}^{\left(k\right)}$. These total tensors are updated recursively at each step *k*:


$$\begin{array}{ccc} {𝐅}^{\left(k\right)} & = & {𝐀}_{k}\cdot {𝐆}_{k}\cdot {𝐀}_{k-1}\cdot {𝐆}_{k-1}\cdots {𝐀}_{2}\cdot {𝐆}_{2}\cdot {𝐀}_{1}\cdot {𝐆}_{1}={𝐀}^{\left(k\right)}{𝐆}^{\left(k\right)}, \end{array}$$ (12a)

$$\begin{array}{ccc} {𝐆}^{\left(k\right)} & = & {\left({𝐀}^{\left(k-1\right)}\right)}^{-1}\cdot {𝐆}_{k}\cdot {𝐀}^{\left(k-1\right)}\cdot {𝐆}^{\left(k-1\right)}, \end{array}$$ (12b)

$$\begin{array}{ccc} {𝐀}^{\left(k\right)} & = & {𝐀}_{k}\cdot {𝐀}^{\left(k-1\right)}. \end{array}$$ (12c)

In this formulation, the incremental growth tensor ${𝐆}_{k}$ is determined by the signaling model via (10). equation (12b) integrates this new growth by first computationally unloading the previous state. Specifically, the inverse of the accumulated elastic tensor, ${\left({𝐀}^{\left(k-1\right)}\right)}^{-1}$, maps the tissue from its current, residually-stressed configuration back to a virtual stress-free state. The new growth increment ${𝐆}_{k}$ is then applied to this relaxed state, and the kinematics are pushed forward again via ${𝐀}^{\left(k-1\right)}$. Finally, the finite element solver computes mechanical equilibrium for this updated total growth tensor ${𝐆}^{\left(k\right)}$, yielding the new incremental elastic tensor ${𝐀}_{k}$, which updates the total elastic deformation via (12c). A step-by-step pseudo-code of this numerical routine is provided in Algorithm A in S1 Appendix.

### Muscle geometries

#### Mesh generation.

Simulations were performed on three distinct types of computational geometries: a cylindrical geometry, an idealized fusiform muscle, and a set of anatomically realistic muscle models (Fig 2F). The idealized fusiform geometry was created to represent a simplified, yet physiologically relevant, muscle shape. The tetrahedral mesh was generated using Gmsh [49] and consists of 1997 cells. The realistic muscle geometries were derived from segmentations of the Visible Human Dataset [39,40]. From these surface representations, tetrahedral meshes were generated using fTetWild [50], resulting in cell counts between 6707 and 9668.

#### Muscle fiber architecture.

Defining the muscle fiber architecture, which sets the local anisotropy ${𝐚}_{0}$, is essential for all geometry types. For the cylinder, fibers simply run parallel along the long axis. In the idealized fusiform model, fibers were defined analytically via a spline curve. For the realistic geometries, fiber directions were estimated by solving a Stokes flow problem [51]. This involved setting the muscle origin and insertion as inflow and outflow boundaries, applying slip conditions on the remaining surface, and normalizing the resulting velocity field to define the local fiber vectors (Fig 2F).

#### Muscle cross-sectional area.

The cross-sectional area (CSA) was computed by integrating over a transverse reference surface $\Gamma_{CS}$ intersecting the muscle at the midpoint of its longitudinal axis. Nanson’s formula [52] provides the mapping from the reference to the deformed area element for this integration:


$$\begin{array}{c} A_{CS}=\int_{\Gamma_{CS}}J\|{𝐅}^{-T}𝐍\|\;dS. \end{array}$$ (13)

For the idealized geometry, $\Gamma_{CS}$ was defined as an internal circular surface located at the geometric midpoint and explicitly included during mesh generation in Gmsh. For each realistic geometry, a unique reference cross-section was defined using principal component analysis (PCA): the primary principal component determined the longitudinal muscle axis, and $\Gamma_{CS}$ was taken as the plane passing through the geometric centroid and normal to this axis. The displacement field was interpolated onto this plane to compute the deformed area.

### Numerical solution schemes and verification

To simulate the coupled mechanics-signaling system, we use a quasi-static approach, justified by the slow timescale of hypertrophy compared to that of elastic deformation [38]. The simulation advances through a series of exercise and rest periods, using a time step of Δt=1 h. Each period begins by solving the signaling ODE system to determine the rate of change in the myofibril population. This growth is then applied incrementally in a nested loop, where each growth step consists of constructing a new growth tensor, solving for mechanical equilibrium, and finally, closing the feedback loop by updating the protein synthesis rate based on the current cross-sectional area (Algorithm A in S1 Appendix). The computational model was implemented in Python, and the simulation code is openly available [53].

The signaling ODE system was integrated using the ‘RK45’ method from SciPy’s solve_ivp function [54], with a local time step of dt = 0.05h. For the spatially varying signaling models, however, a custom implementation of the fourth-order Runge–Kutta (RK4) method was used. We use the finite element method to numerically solve the mechanical equilibriumnonlinear equations (3), using the FEniCSx finite element software [55]. Relative to each of the computational meshes, we define the finite element space 𝐕 as the space of continuous piecewise quadratic 3-vector fields and *Q* as the space of continuous piecewise linears. We then introduce the test functions 𝐯∈𝐕 and q∈Q. At each iteration, we solve the following system of nonlinear equations: find the approximate displacement 𝐮∈𝐕 and pressure p∈Q solving


$$\begin{array}{cc} \int_{\Omega }𝐏:\nabla 𝐯\;d𝐗-\int_{\Gamma_{R}}{𝐓}_{R}\cdot 𝐯\;d𝐒 & =0\;\;\forall \;𝐯\in 𝐕, \end{array}$$ (14a)

$$\begin{array}{cc} \int_{\Omega }q(J_{e}-1)\;d𝐗 & =0\;\;\forall \;q\in Q, \end{array}$$ (14b)

where 𝐏=𝐅𝐒 is the first Piola–Kirchhoff stress tensor, with 𝐒 defined in (1). The nonlinear system (14) was solved using a Newton solver. To verify the implementation, we performed a series of numerical tests, including evaluating the correctness of the numerical solution algorithms for the spatially varying ODE systems (Fig A in S1 Appendix) and a mesh and time step convergence test for the full growth simulation (Fig B in S1 Appendix).

## Results

### Signaling model links exercise protocol to distinct dynamic responses

Before implementing the full multiscale framework, we first characterized the dynamics of the core signaling model in isolation. We simulated the model’s response to three distinct exercise protocols (Fig 2C): (1) a high-intensity, high-frequency protocol (MWF); (2) a high-intensity, low-frequency protocol (weekly); and (3) a low-intensity, medium-frequency protocol (every three days).

**Fig 3:**
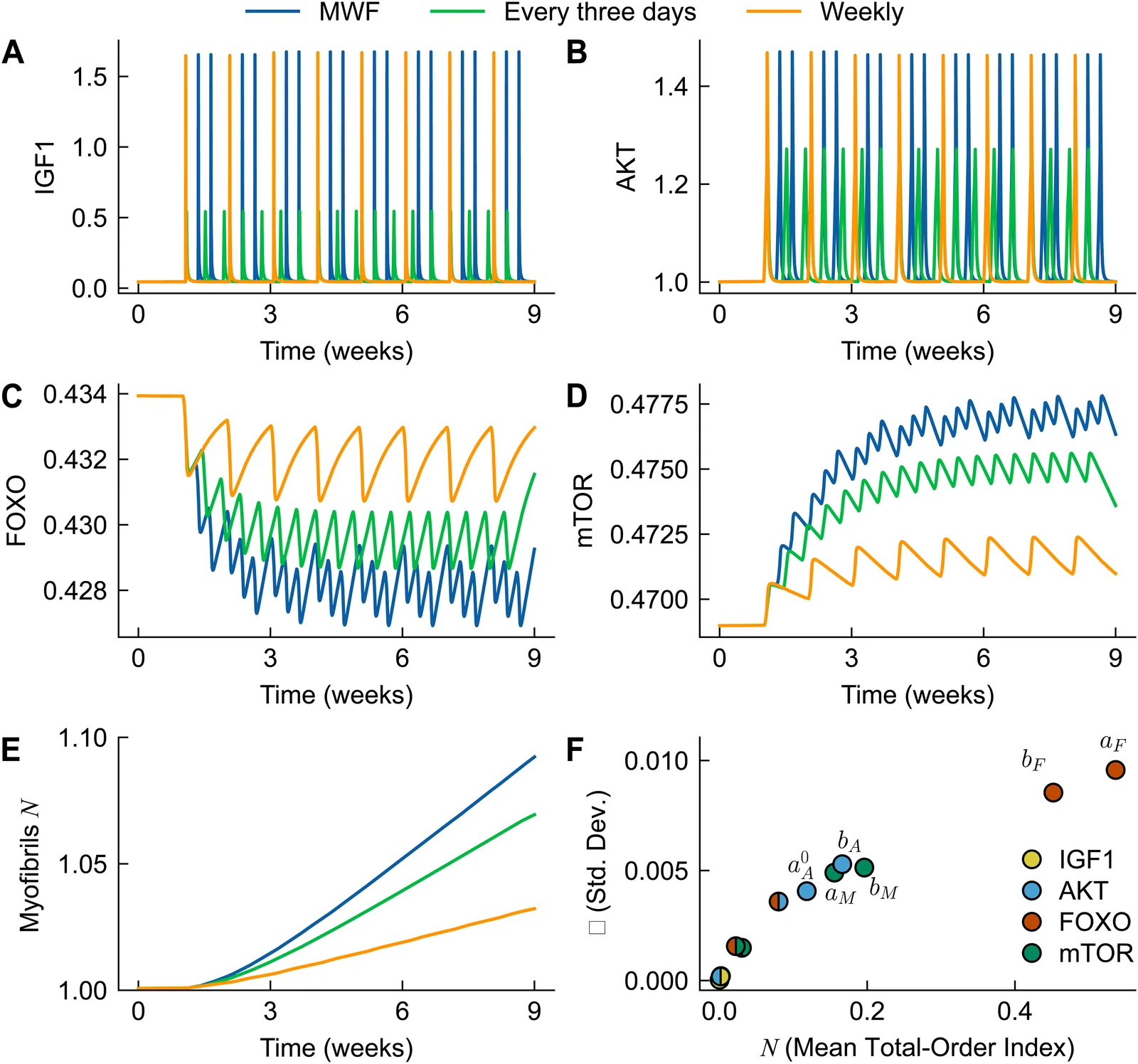
Dynamics of the IGF1-AKT signaling model in response to different exercise protocols. **(A–E)** Time courses of the normalized populations **(A)** IGF1, **(B)** AKT, **(C)** FOXO, (**D**) mTOR, and (**E**) myofibrils *N* for three different exercise protocols. Insets in (**A**) and (**B**) display a zoomed-in 24-hour time window to highlight the rapid transient spikes in IGF1 and AKT signaling. **(F)** Sobol sensitivity analysis quantifying the influence of each model parameter on the final myofibril number obtained using a standardized daily exercise protocol. The plot shows the mean Sobol index versus its standard deviation for each parameter. Marker colors identify the associated signaling molecule, with split colors indicating coupling parameters.

The model exhibited a dynamically consistent response across all protocols (Fig 3A-3E). Each exercise bout triggered a rapid, transient spike in the upstream signals IGF1 and AKT, with a peak observed approximately 12 hours post-stimulus (Fig 3A-3B). The amplitude of these spikes was directly proportional to the exercise intensity. The upstream activation drove the expected opposing response in the downstream effectors: mTOR levels (promoting synthesis) increased, while FOXO levels (promoting degradation) were suppressed (Fig 3C-3D). Importantly, the system operates on two distinct timescales. IGF1 and AKT act as fast responders, returning to baseline between each exercise period. In contrast, FOXO and mTOR display a wave summation effect, wherein the responses to repeated stimuli accumulate and oscillate around an apparent steady state. The magnitude of this shift is protocol-dependent and directly determines the final myofibril density *N* (Fig 3E).

To identify which model parameters were most critical in controlling this response, we performed a Sobol sensitivity analysis. The analysis revealed that the final myofibril population was most sensitive to parameters governing the downstream effector, FOXO (Fig 3F). Specifically, the intrinsic growth ($a_{F}$) and self-inhibition ($b_{F}$) rates of FOXO were the most influential, followed by the corresponding rates for mTOR ($a_{M}$ and $b_{M}$) and AKT ($a_{A}^{0}$ and $b_{A}$). This sensitivity arises because the core signaling network functions as an incoherent feedforward loop [24,25]: the upstream signal (AKT) simultaneously promotes a synthesis pathway (mTOR) and suppresses a degradation pathway (FOXO). Consequently, the qualitative outcome – whether the muscle undergoes hypertrophy or atrophy – is not determined by the exercise protocol alone but depends critically on the kinetic balance between these opposing branches. The high sensitivity to FOXO parameters indicates that $a_{F}$ and $b_{F}$ effectively set a threshold for growth. Small perturbations to these rates can abruptly toggle the net protein turnover from positive to negative, even under identical exercise stimuli.

Comparing the net growth response between the regimens, the high-intensity, high-frequency (MWF) protocol induced the largest increase in the myofibril population (*N*) due to the summation effects described above (Fig 3E). However, all exercise protocols predicted net growth relative to the sedentary baseline, confirming that even lower-frequency stimuli provide a sufficient perturbation to shift the net protein balance. With these protocol-dependent trajectories of *N* established, we next used them as the driving input for the full 3D framework to investigate how biochemically driven growth manifests as mechanical remodeling across idealized muscle geometries.

### Coupled model predicts protocol-dependent hypertrophy in an idealized muscle geometry

**Fig 4:**
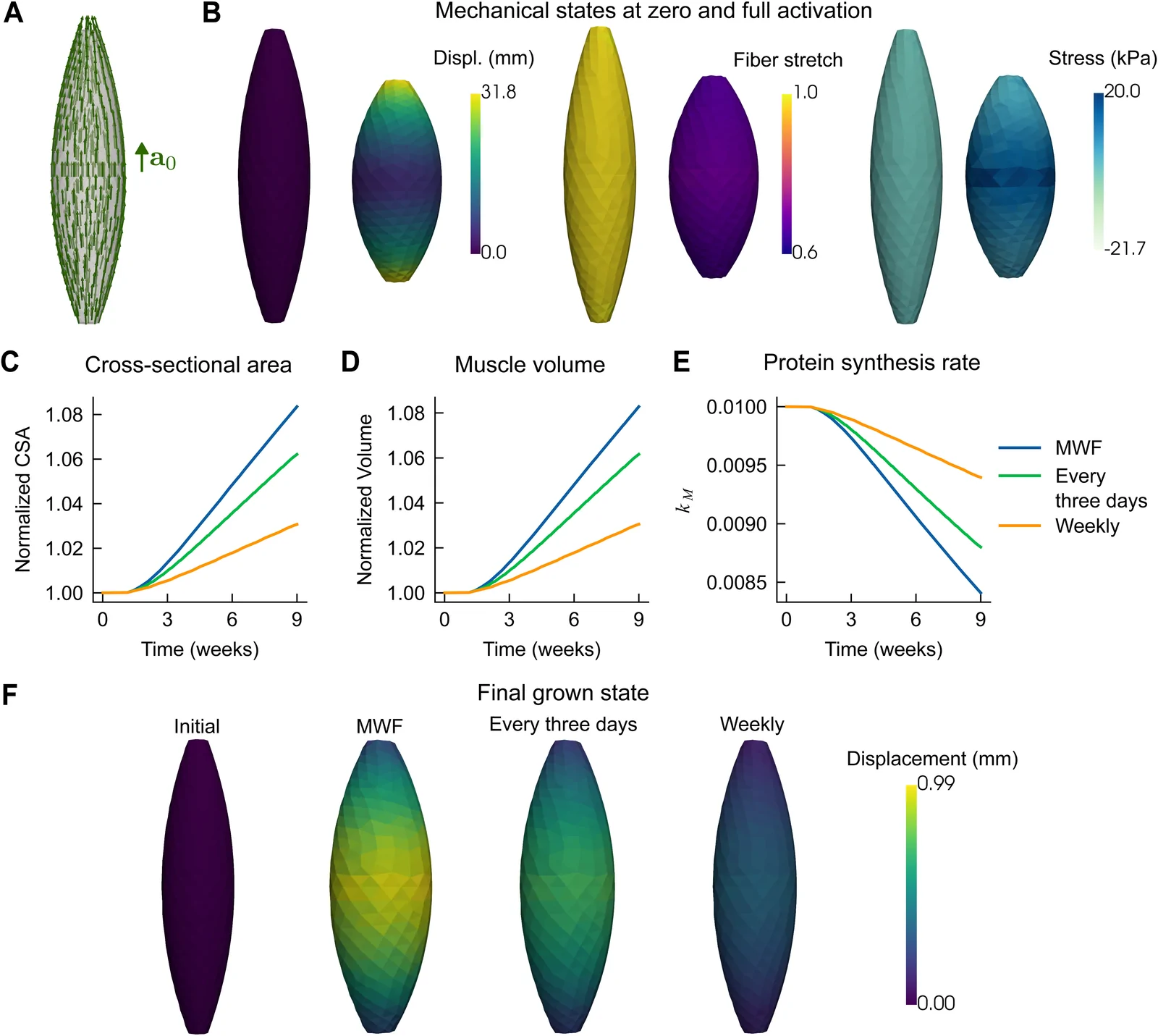
Coupled model simulation of exercise-induced hypertrophy in an idealized fusiform muscle. **(A)** Fiber orientation *a*_0_ computed from a spline expression. **(B)** Mechanical state variables (displacement, fiber stretch λ, and fiber stress) at zero and full activation (α=1). **(C–E)** Predicted time courses of cross-sectional area, muscle volume, and the protein synthesis rate $k_{M}$ in response to three distinct exercise protocols. **(F)** Final muscle configurations after the simulation period for each protocol, with the growth-induced displacement amplified 10x for visualization.

While biochemical signaling and tissue mechanics are often modeled in isolation, physiological growth is an inherently coupled phenomenon. Accordingly, we next integrated the signaling model with the 3D hyperelastic framework, initially simulating long-term adaptation in an idealized fusiform muscle geometry (Fig 4A).

First, we characterized the muscle’s baseline mechanical behavior in the absence of volumetric growth. A simulated contraction from zero to full activation (α from 0 to 1) produced heterogeneous distributions of displacement, stress and fiber stretch along the muscle geometry (Fig 4B). Muscle ends were allowed to partially contract towards the midplane. The displacement magnitudes were largest at the ends of the muscle, consistent with this shortening behavior. Tensile fiber-direction Cauchy stress peaked at the surface of the muscle center, transitioning to negative (compressive) values internally, whereas the fiber stretch λ was relatively uniformly distributed, exhibiting only slightly higher values in the central region.

Having established that the mechanical framework gives the expected response for an idealized muscle geometry, we next combined the signaling model with the muscle growth model. We simulated muscle adaptation over a nine-week period for the three different exercise protocols. The coupled model successfully translated the protocol-dependent signaling dynamics into different macroscopic growth in an exercise-dependent manner. Consistent with the myofibril-level predictions, the high-intensity, high-frequency MWF protocol induced the most significant increase in both cross-sectional area (CSA) and total muscle volume, followed by the every-three-days and weekly protocols (Fig 4C-4E). Furthermore, we also identified the critical role of homeostatic control generated by the feedback between signaling and mechanics. As the muscle CSA increased in response to training, the mechanical feedback loop progressively downregulated the protein synthesis rate, $k_{M}$, from its baseline value (Fig 4C, 4E). The strength of this feedback is dependent on the exercise modality – higher exercise frequency leads to a sharper drop in the protein synthesis rate, naturally limiting the rate of further hypertrophy (Fig 4D). The macroscopic effect of this adaptation is visualized in Fig 4F, which shows the final deformed muscle shapes for each protocol (growth amplified tenfold for visualization). This result is a direct consequence of the anisotropic growth tensor included in the model (10), which mechanistically links the signaling output to mass addition in the plane perpendicular to the fiber axis. Overall, these simulations suggest that while any exercise stimulus promotes growth relative to the baseline, the mechanical feedback loop prevents a linear dose-response, ensuring the higher-frequency training leads to self-limiting adaptation rather than unbounded hypertrophy.

### Anatomically realistic models show protocol-dependent adaptation and non-uniform growth

**Fig 5:**
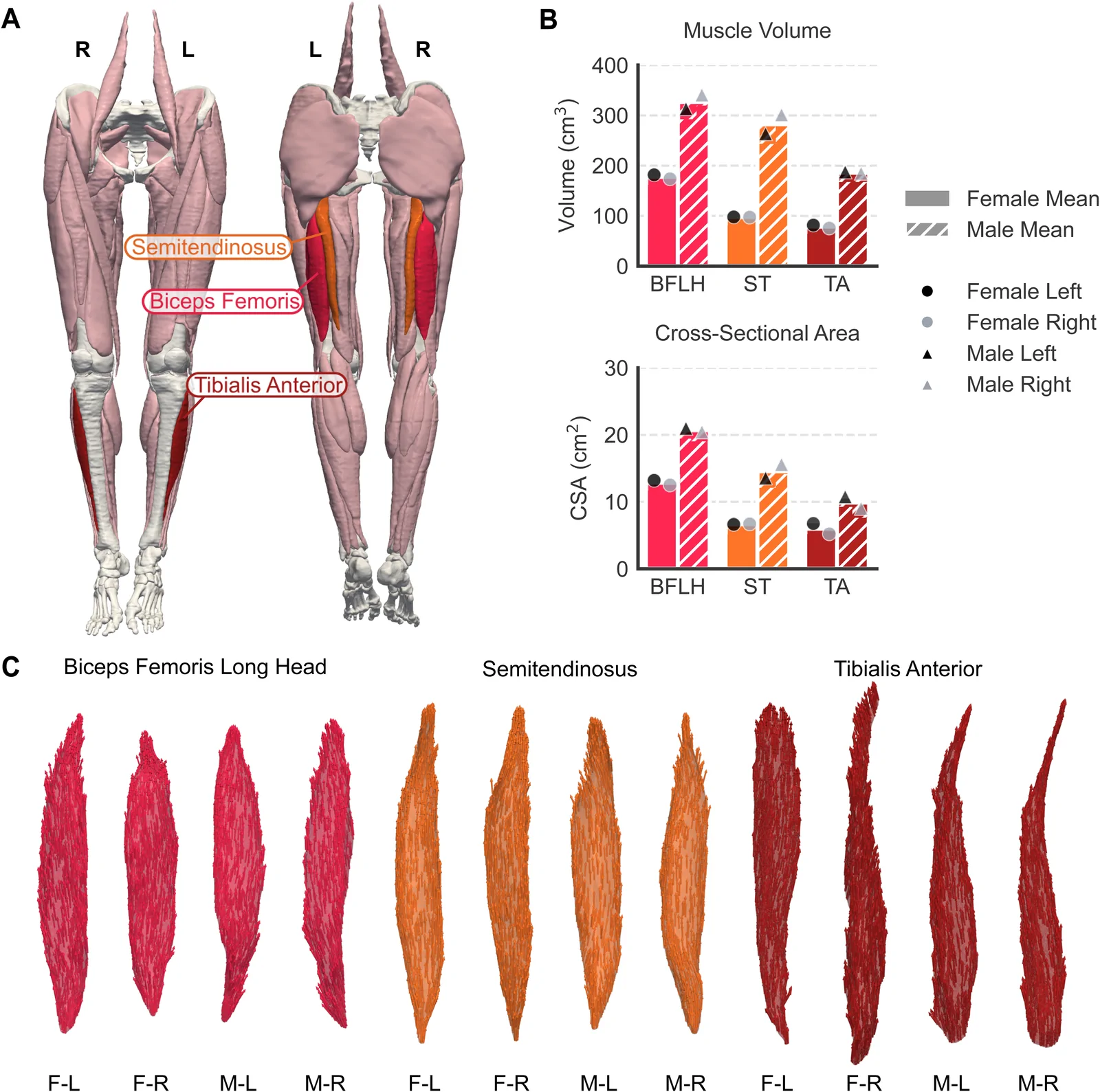
Realistic muscle geometries derived from the Visible Human dataset. **(A)** Anatomical placement of the semitendinosus (ST), biceps femoris long head (BFLH), and tibialis anterior (TA) muscles, visualized within the Visible Human Female. **(B)** Comparison of computed muscle volumes and cross-sectional areas for the twelve meshes derived from the bilateral ST, BFLH, and TA muscles of the Visible Human Female and Male. **(C)** Fiber arrangements generated using Stokes flow simulations within meshes derived from the surface geometries shown in **(A)**. Labels indicate gender (F: Female, M: Male) and laterality (L: Left, R: Right).

Thus far, we used idealized geometries to investigate how muscle growth responds to different exercise modalities. To determine how complex, subject-specific anatomy influences this response, we next applied the coupled framework to a cohort of 12 anatomically realistic muscle geometries derived from the Visible Human Dataset [39,40]. The cohort consisted of bilateral (left and right) meshes for both a male and female subject, yielding four unique geometries for each of the three selected muscle types: the biceps femoris long head (BFLH), semitendinosus (ST), and tibialis anterior (TA) (Fig 5A). Functionally, the BFLH and ST act as hamstring knee flexors and hip extensors, while the TA serves as a primary ankle dorsiflexor. These muscles are heavily recruited during common activities such as running, squatting, and lunging, making them highly relevant for investigating exercise-induced adaptation. Prior to simulation, we characterized the baseline geometries of the cohort, confirming that male muscles displayed larger initial volumes and cross-sectional areas compared to their female counterparts (Fig 5B). Furthermore, to capture the internal architecture essential for anisotropic growth, we generated subject-specific fiber arrangements for each mesh using Stokes flow simulations (see Methods, Fig 5C, and Fig F in S1 Appendix).

**Fig 6:**
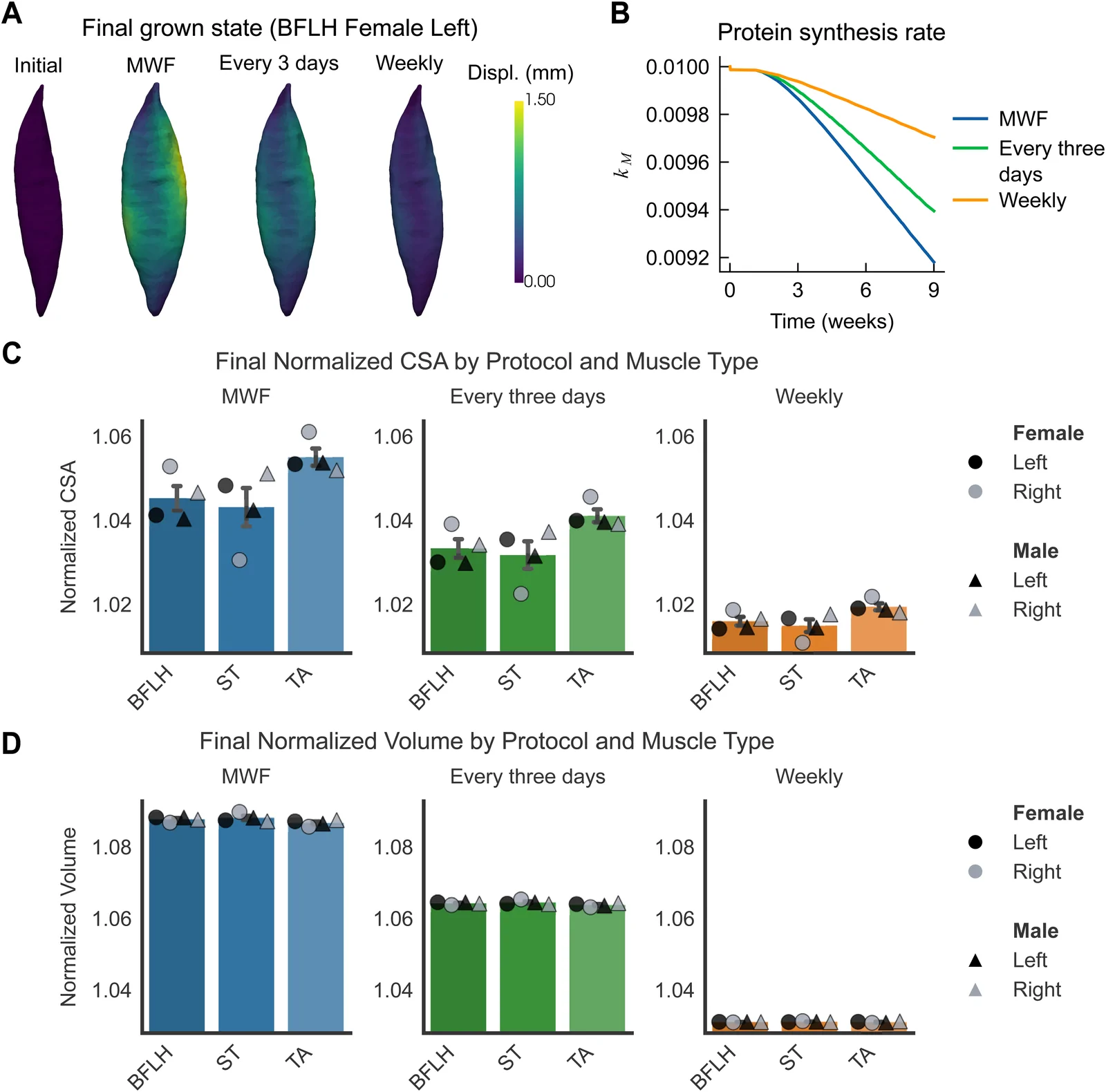
Coupled model simulation of exercise-induced hypertrophy in realistic muscle geometries. **(A)** Final muscle configurations of the female left biceps femoris long head (BFLH) following the simulation period for each exercise protocol. Growth-induced displacements are amplified 10x for visualization. **(B)** Predicted temporal evolution of the protein synthesis rate ($k_{M}$) in response to three distinct loading protocols (shown for the female left BFLH geometry). **(C–D)** Predicted final CSA and volume after three protocols in the BFLH, ST, and TA muscles. Error bars indicate standard error.

We first examined the model’s predictions for a representative case: the female left biceps femoris long head (BFLH). The 3D visualizations of the muscle following the nine-week training protocols reveal a distinct, protocol-dependent hypertrophic response (Fig 6A). Consistent with the high-intensity nature of the stimulus, the MWF protocol produced the most substantial visible thickening of the muscle belly, followed by the every-three-days and weekly protocols. Our simulations show that the complex, subject-specific architecture results in irregular tissue expansion, in contrast to the symmetric radial scaling observed in the idealized model. While the idealized geometry distributes growth evenly around the central axis (Fig 4F), the realistic geometry exhibits spatially heterogeneous hypertrophy, with peak displacement localized to specific regions of the muscle belly. To understand the regulation underlying this growth, we tracked the temporal evolution of the protein synthesis rate, $k_{M}$ (Fig 6B). The simulations show a progressive downregulation of $k_{M}$ from its baseline value as the tissue adapts. This decay reflects the homeostatic feedback loop: as the muscle grows, the mechanical stimulus for further synthesis is reduced, naturally limiting the adaptation rate over time.

Expanding this analysis to the full cohort of 12 geometries, we found that these protocol-dependent trends were robust across all subjects and muscle types. For every geometry tested, the high-frequency, high-intensity MWF protocol induced the most pronounced hypertrophy, followed by the every-three-days and weekly protocols (Fig 6C-6D). A key finding from the cohort analysis was that the relative increase in total muscle volume was consistently larger than the relative increase in cross-sectional area (CSA) measured at the muscle’s midplane. For instance, the MWF protocol induced an average volume increase of approximately 8–9% (Fig 6D), while the corresponding CSA increase was only 3–6% (Fig 6C). This discrepancy arises because the complex architecture drives non-uniform growth, where mass is added asymmetrically along the muscle. Consequently, site-specific CSA measurements, a common metric in clinical and experimental studies [56], may systematically underestimate the extent of tissue adaptation. The variability was notably more pronounced in the normalized CSA measurements (Fig 6C) compared to the more tightly clustered volume measurements (Fig 6D). This reinforces that CSA is a localized metric, highly sensitive to anatomical placement and local geometric irregularities, whereas volume more robustly captures the global adaptation. Within the scope of our simulations, we did not see any muscle type specific or sex specific differences in response to exercise.

In conclusion, these results demonstrate that tissue geometry and fiber architecture play a fundamental role in the spatial distribution of hypertrophy. Because the model defines growth as mass addition transverse to the local fiber axis, the subject-specific fiber orientation directly dictates the direction of expansion at every point. Consequently, complex fiber arrangements result in non-uniform shape changes that cannot be fully captured by idealized models, highlighting the need for anatomical fidelity in predictive modeling of muscle adaptation.

### Tissue mechanics integrates heterogeneous signaling into smooth deformation

Finally, to mimic cell-to-cell variability in muscle tissue, we investigated how inherent spatial variation in biochemical signaling affects the mechanochemical response to exercise. Moving beyond the assumption of homogeneous tissue properties, we conducted an ensemble analysis on the female left BFLH geometry using the high-intensity MWF protocol. This specific combination was selected as the “maximal response” case to determine if robust hypertrophy persists even under noisy signaling conditions. In these simulations, all rate parameters associated with IGF1, AKT, FOXO, and mTOR were simultaneously perturbed by ±1% using a uniform random distribution at each mesh cell. Crucially, given the incoherent feed-forward loop structure of the signaling network, such parameter fluctuations have the potential to qualitatively alter the local outcome, flipping the kinetic balance from net synthesis to degradation.

**Fig 7:**
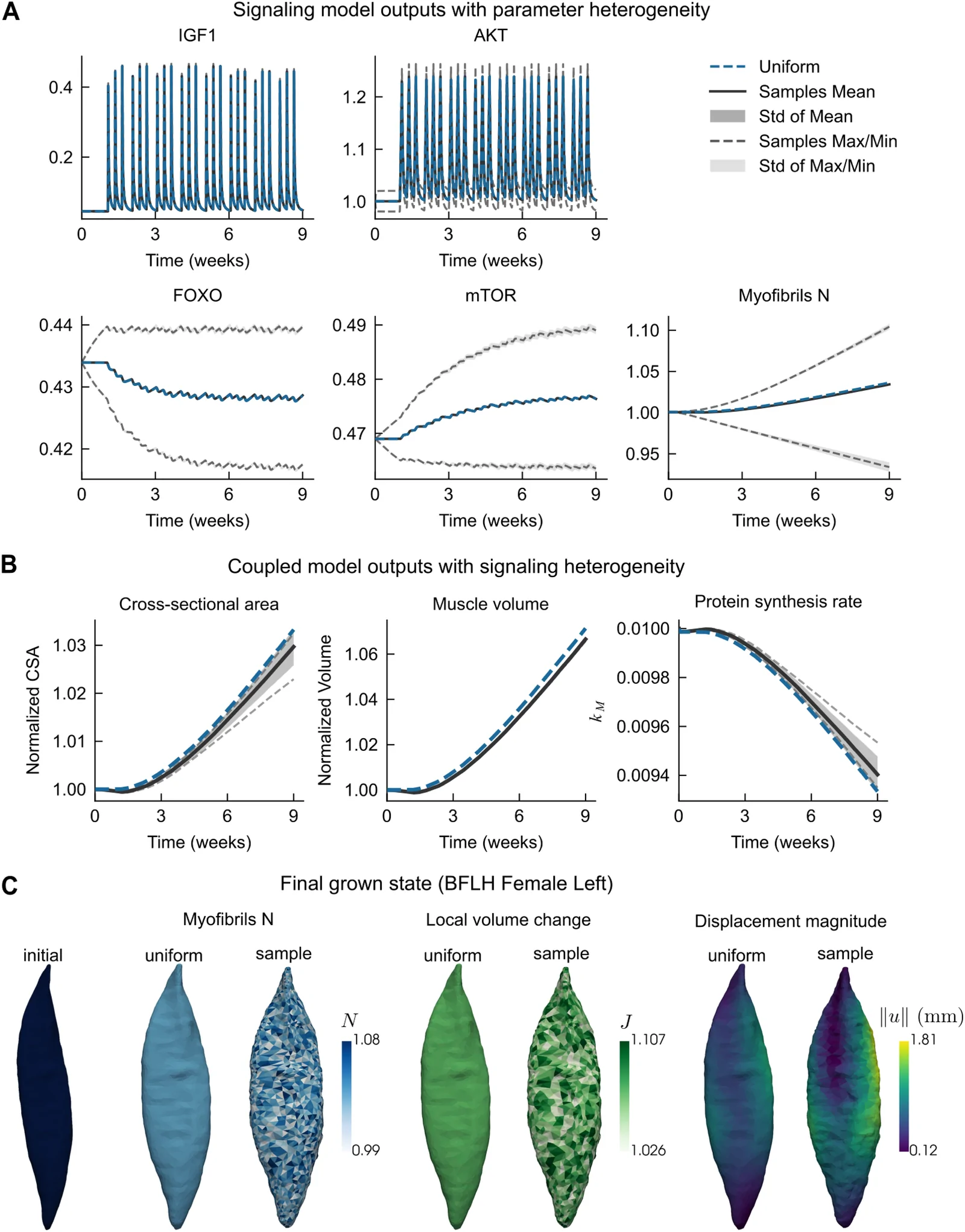
Impact of signaling parameter heterogeneity on local and global growth dynamics. **(A)** Predicted time courses of the local ODE states (IGF1, AKT, FOXO, mTOR, and myofibril population N). Heterogeneity was introduced by sampling rate parameters ($a_{i}$, $b_{i}$, $c_{ij}$) from uniform distributions within ±1% of their baseline values. Gray lines and shading indicate the mean ±1 standard deviation across five samples; the dashed blue line represents the baseline uniform case. **(B)** Predicted evolution of normalized cross-sectional area (CSA), normalized volume, and the protein synthesis rate $k_{M}$ under the MWF protocol (colors as in **A**). **(C)** Spatial maps of final myofibril density (N), local volume change (*J*), and growth-induced displacement magnitude (amplified 10x) for the uniform case versus a representative heterogeneous sample.

The time courses of the local ODE states reveal the model’s sensitivity to cellular variability (Fig 7A). While the ensemble’s mean signaling response remained consistent with the uniform baseline, the variance was significantly amplified in the downstream regulators, FOXO and mTOR. This amplification reflects a key biological feature: because downstream molecules integrate signals over time, they accumulate and magnify small upstream fluctuations. Consequently, even under the optimal high-intensity (MWF) stimulus, local outcomes vary significantly. Although the average response is positive, specific regions may experience blunted growth or even atrophy due to unfavorable parameter combinations.

At the macroscopic level, the uniform baseline case consistently achieved more total growth than the mean of the heterogeneous ensemble, as measured by both normalized volume and CSA (Fig 7B). This indicates that the introduction of signaling noise creates a net drag on the system, where the cumulative effect of suboptimal local responses slightly dampens the aggregate adaptation compared to the idealized, uniform condition. This difference in growth is reflected in the protein synthesis rate, $k_{M}$. Since the uniform case achieves a larger increase in CSA, it triggers a correspondingly larger decrease in $k_{M}$ compared to the heterogeneous average. Furthermore, this analysis confirmed our previous observation that variability was consistently greater for normalized CSA than for total volume. Because CSA is a localized slice, it is highly susceptible to specific regional fluctuations, whereas total volume integrates over the entire domain, effectively smoothing out the local noise to provide a more robust metric of adaptation.

The 3D spatial maps reveal a striking contrast between the biological drive and the mechanical response (Fig 7C). The heterogeneity in the myofibril population (*N*) is closely reflected in the speckled, fragmented map of local growth (*J*). However, the resulting displacement magnitude shows a smoother and more coordinated pattern. This phenomenon demonstrates a mechanical buffering, where the global equilibrium constraints of the tissue force the continuum to deform smoothly. Effectively, the elastic matrix averages out the highly variable local expansion rates, preventing individual cells from expanding or shrinking in isolation. However, this buffering comes at a cost. The mismatch between the jagged local growth drive (*J*) and the smooth deformation gradient implies the generation of internal residual stresses. It is possible that regions of muscle tissue attempting to grow rapidly are physically constrained by their slower-growing neighbors, creating a mechanical drag that not only dampens the aggregate expansion but may also generate localized shear concentrations prone to micro-damage.

## Discussion

In this work, our goal was to develop a multiscale computational framework to quantify the effects of different exercise modalities on realistic human muscle geometries. This framework successfully integrated IGF1-AKT signaling dynamics with 3D hyperelastic tissue mechanics to model long-term skeletal muscle hypertrophy. We first established that the readouts of the signaling pathway are highly sensitive to the exercise protocol, with the high-intensity, high-frequency regimen inducing the most significant growth. The architecture of the signaling network resembles an incoherent feedforward loop [24,25]. We identified FOXO and mTOR as the critical points governing the balance between protein synthesis and degradation for muscle hypertrophy, though further experimental validation is required to confirm this. The same stimulus resulting in a balance of synthesis and degradation is a common feature found in many signaling pathways [57–59] and is important for maintaining homeostasis. Applying the coupled model to anatomically realistic human geometries confirmed this protocol-dependent adaptation, highlighting that the feedback between mechanical deformation and signaling is essential for regulating the magnitude of hypertrophy and stabilizing the growth process. Our simulations also revealed that the relative increase in total muscle volume consistently exceeds the increase in cross-sectional area (CSA) in realistic geometries. Finally, our analysis of spatial heterogeneity revealed that while local volume expansion rates are highly localized by biological variability, the tissue-wide integration of mechanics acts as a buffer, forcing the overall displacement and resulting bulk shape change to remain relatively smooth and coordinated.

While our current framework lacks the matched longitudinal data required for a rigorous quantitative validation, its outputs fall within physiologically plausible orders of magnitude when compared to anatomical and physiological benchmarks. First, the baseline muscle geometries derived from the Visible Human dataset are consistent with MRI-derived population averages. For instance, Handsfield et al. [60] reported mean volumes of 206.5 cm^3^ for the BFLH, 186.0 cm^3^ for the ST, and 135.2 cm^3^ for the TA. These values compare favorably with our model’s initial volumes of 252.4 cm^3^, 190.1 cm^3^, and 132.4 cm^3^, respectively, supporting the anatomical realism of the meshes but not validating the growth model itself. Regarding the link between mechanical workload and biological response, our model successfully predicted the dose-dependent nature of hypertrophy. We observed that myofibril accretion is highly sensitive to the exercise regimen, with high-frequency, high-intensity protocols inducing the steepest monotonic increase. This prediction aligns with the systematic review by Schoenfeld et al [61], which identifies a significant positive relationship between weekly training frequency (in the range 1-3x/week) and muscle growth. Building on this biochemical foundation, the predicted magnitude of macroscopic hypertrophy falls within a plausible range compared to existing experimental literature. For example, our simulation of the DeFreitas (MWF) protocol on realistic geometries predicted a total volume increase of nearly 9%. Although not a direct validation, as it involves a different muscle group and experimental context, this is on the same order of magnitude as the 10.1-11.3% volume increase reported by Kubo et al [62] in the pectoralis major following 10 weeks of training. Interestingly, the associated increase in mid-belly cross-sectional area was more conservative (~ 4–6%) than the range observed in comparable resistance training studies. Specifically, our predictions are slightly lower than the 9.6% increase in thigh muscle CSA reported by DeFreitas et al [45], the 10% increase found by Damas et al. [63] in vastus lateralis after 10 weeks of resistance training, and the 7.4% increase reported by Seynnes et al [64] in the quadriceps femoris following a five-week high-intensity resistance training program. Unlike idealized geometries where volume scales predictably with CSA (Fig 4C, 4D), anatomical muscles exhibit spatially heterogeneous growth, such that local CSA changes may not directly reflect whole-muscle hypertrophy. Thus, our results suggest that single-slice CSA measurements may underrepresent the total hypertrophic response in complex muscle shapes. This may partly explain why our predicted CSA increases are smaller than those reported experimentally. However, the discrepancy may also reflect deficiencies in the current framework. The underprediction of CSA may stem from the highly reduced nature of the signaling model or the use of generalized material parameters that do not capture the exact biomechanical behavior of the specific muscle types studied. A rigorous quantitative validation of the coupled model will require longitudinal datasets suitable for parameter calibration and independent testing, which were not available for the present study.

While the proposed framework captures key mechanistic links between signaling and tissue mechanics, we note some limitations and opportunities for future computational development. First, the signaling model is a reduced representation of the underlying biochemical network and omits additional pathways and fast-timescale dynamics. A complex signaling model could capture more biochemical interactions, but at the cost of higher computing demands and the introduction of parameters that are hard to estimate [65]. A future modeling direction could involve coupling detailed signaling models [28,66,67] with multicompartmental, mixed-dimensional PDE growth models [68] to represent different length scales from subcellular organelles to muscle cross-section. Our framework also combines biochemical and mechanical models developed from different sources. Although this integration enables a comprehensive computational framework, establishing parameter provenance and confidence across coupled models remains a long-standing challenge in computational and systems biology that will require concerted effort across the field to resolve [69,70]. Second, we model growth as transverse expansion driven by a global signaling output, rather than by localized mechanical stimuli (e.g., tissue stress or strain). As a result, the aggregate 0D exercise parameters ($I_{ex}$, $F_{\max}$) are completely decoupled from the parameters of the continuum model (α, $\sigma_{active}$). Relating these time-averaged, macroscopic variables to local mechanotransduction pathways remains an open challenge for future modeling. Furthermore, our model does not account for along-fiber remodeling, known as sarcomerogenesis [71]. This means the model captures how the tissue thickens (hypertrophy) but misses how fibers lengthen to adapt to chronic stretch, an important aspect of tissue remodeling. Accurate representation of either growth mechanism ultimately depends on the underlying fiber architecture, since local fiber orientation dictates the axis of both force generation and tissue expansion. Here, fiber paths were estimated using Stokes flow, which provides a mathematical idealization that enforces a generalized alignment from origin to insertion but cannot capture more complex architectures such as the bipennate structure of the tibialis anterior (TA). These generated fiber fields should therefore be viewed as computational proxies rather than high-fidelity anatomical reconstructions. Future improvements could incorporate liquid-crystal models, as recently demonstrated for cardiac muscle [72], while the framework is also readily extensible to patient-specific fiber architectures derived from diffusion tensor imaging (DTI) as such data become more available. Nevertheless, the current cohort provides a valuable testbed for demonstrating the framework’s sensitivity to 3D spatial heterogeneity and its potential for personalized simulations. The mechanical model also remains simplified in several aspects. Incorporating muscle-specific material parameters as experimental measurements become available, for instance, in the force-length relationships, could improve predictive capability. Likewise, the boundary conditions employ linear elastic springs primarily to ensure computational stability rather than to faithfully represent tendon mechanics. Future work could instead incorporate patient-specific tendon and aponeurosis geometries, enabling more realistic nonlinear tissue compliance. Finally, assigning independent random values to each element makes the spatial heterogeneity length scale mesh-dependent. We adopt this uncorrelated distribution as a theoretical stress test to demonstrate the capacity for mechanical buffering. Since actual biological heterogeneity likely manifests as smoother, spatially correlated fields, we expect the qualitative buffering effect to remain, although its quantitative characteristics may differ.

This study establishes a modular computational infrastructure for linking cellular biology to organ-level morphology. A key feature of this mechanochemical framework is its extensibility. The reduced IGF1-AKT model serves as an adaptable component that can be substituted with more expansive systems biology models to capture phenomena such as metabolic fatigue, calcium handling, or gene regulation. On the mechanical side, the kinematic description of growth can be extended to include sarcomerogenesis [73]. Incorporating this mechanism in future iterations could enable the differentiation between concentric-induced thickening and eccentric-induced fascicle lengthening, an important capability for analyzing diverse training modalities. A critical next step will be closing the loop on mechanochemical coupling by transitioning from global signaling outputs to local mechanical drivers. Moreover, developing the computational strategies necessary to quantify accumulated residual stresses, alongside evaluating additional metrics like pennation angle, curvature, or physiological CSA, could provide a more comprehensive characterization of the structural adaptations than simple macroscopic volume and anatomical CSA. Beyond mathematical extensions, our framework lays the groundwork for integrating the next generation of complex anatomical data. With the capability to model growth on realistic geometries established, there is a need for larger geometric cohorts and subject-specific fiber architectures derived from DTI. Our platform is ready to incorporate such datasets as they become available, allowing for the rigorous characterization of how anatomical variability dictates functional adaptation. Ultimately, by bridging the gap between molecular drivers and macroscopic function, our computational framework provides the foundation for simulating individual-specific scenarios, from optimizing athlete peak performance to designing targeted rehabilitation protocols for muscle atrophy.

## Supporting information

S1 AppendixFig B. Growth simulation verification.Fig C. Parameter fit of the protein synthesis rate $k_{M}$. Fig D. Comparison of Hill-type, linear, and no feedback. Fig E. Illustration of reduced growth rate due to feedback. Fig F. Illustration of muscle fiber directions. Algorithm A in S1 Appendix. Cumulative growth implementation.(PDF)
