# Supplementary material for: Mechanochemical modeling of exercise-induced skeletal muscle hypertrophy
Source: PLoS Comput Biol. 2026 Aug 24;22(8):e1014691. doi: 10.1371/journal.pcbi.1014691 (PMC13529250; doi:10.1371/journal.pcbi.1014691)
Supplement: Supplementary file 1 [file pcbi.1014691.s001.pdf]

S1 Appendix

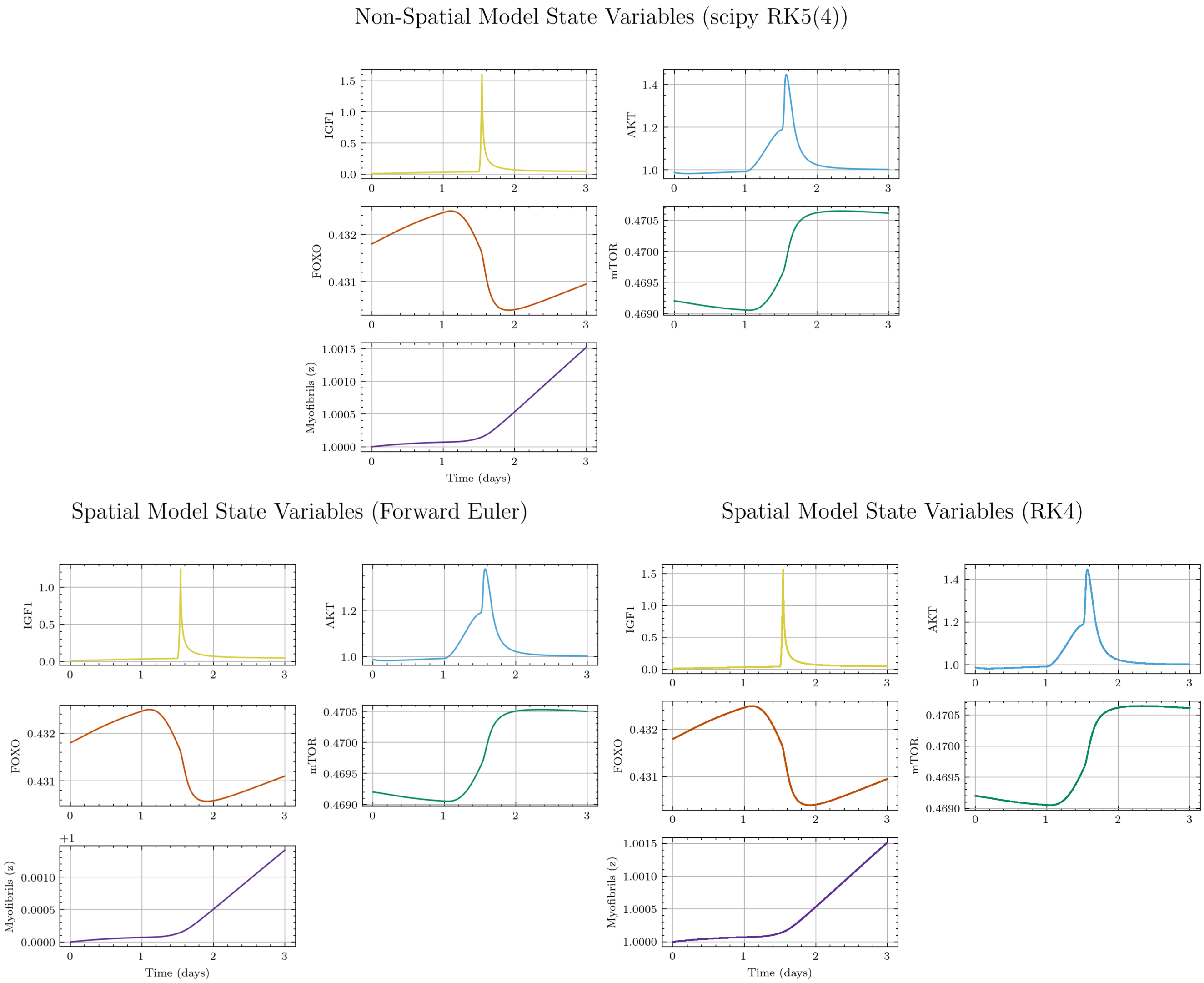

Fig A. ODE solver verification. Time course of the ODE state variables comparing the non-spatial SciPy RK45 reference and spatial integrators (forward Euler, RK4) for a single exercise session with homogeneous parameters and time step $\Delta t = 0.05$ h. The spatial RK4 closely matches the RK45 solution.

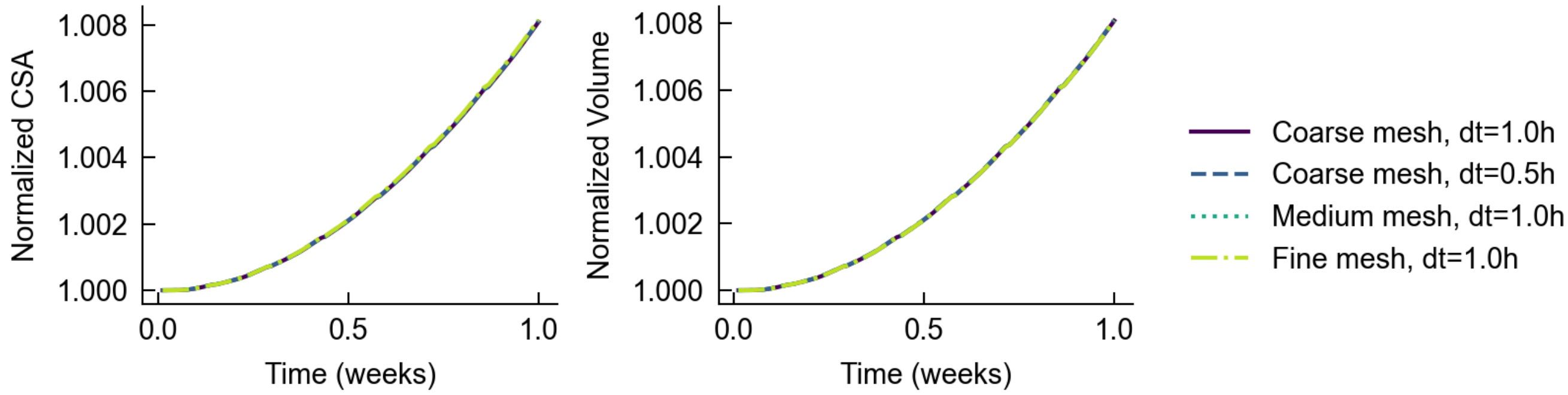

Fig B. Growth simulation verification. Time course of cross-sectional area and muscle volume for a one-week exercise protocol with daily 1-hour exercise. We use three uniform mesh refinement levels of the idealized fusiform geometry, with 1997 ("Coarse mesh"), 15976 ("Medium mesh") and 127808 ("Fine mesh") cells, respectively. For the coarse mesh, we also do one time step refinement, reducing the growth time step from $dt = 1$h to $dt = 0.5$h. The results show no visible difference between the four cases. The symmetric relative difference in final CSA between the coarsest and finest mesh is on the order of 0.005%.

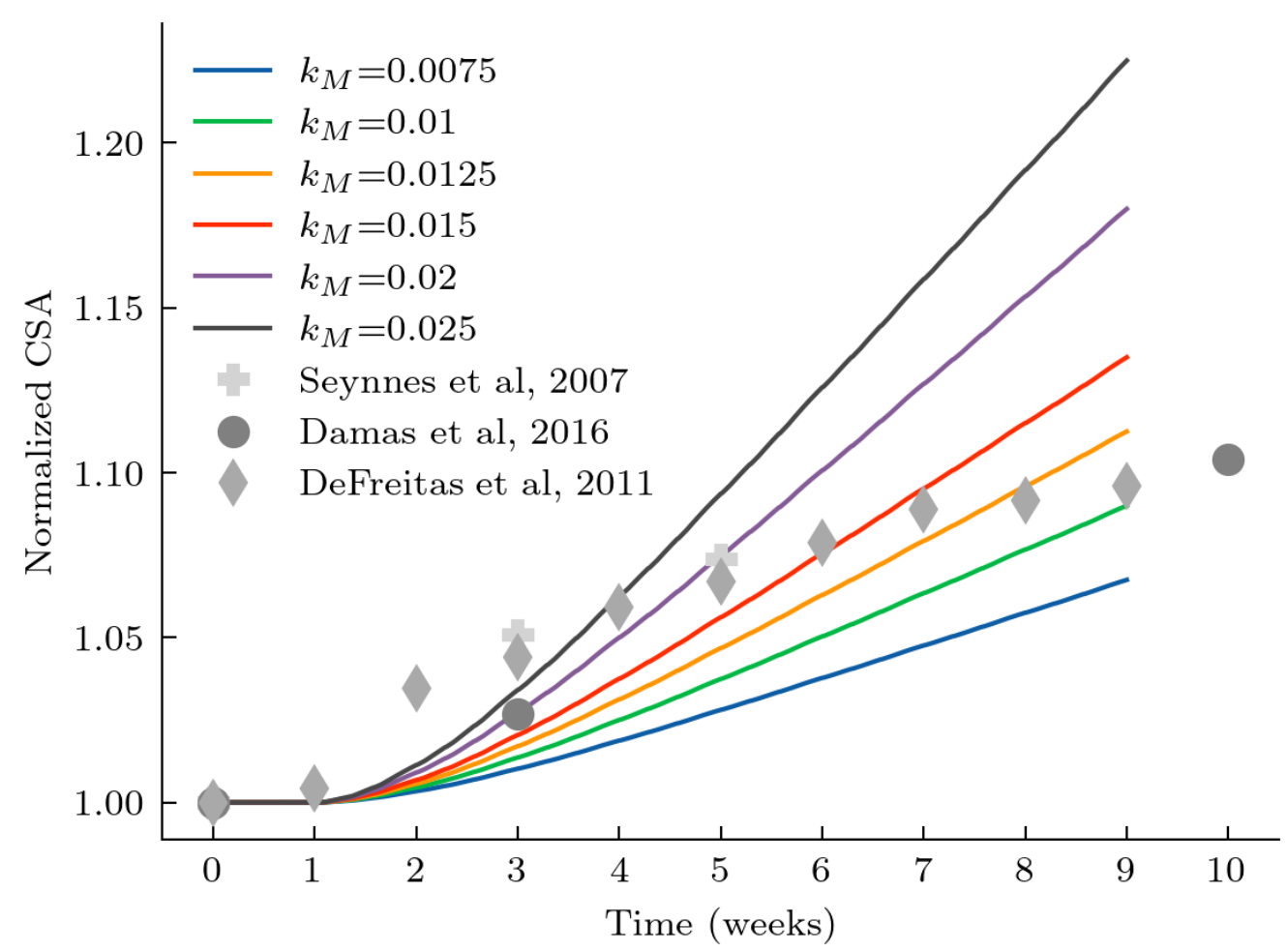

Fig C. Parameter fit of the protein synthesis rate k_M. To verify the model's physiological relevance, we compared the simulated growth against experimental cross-sectional area (CSA) data. The plot shows the cross-sectional area (CSA) over time for different values of the protein synthesis rate k_M, for the DeFreitas (MWF) exercise protocol. The results are compared to experimental data from DeFreitas et al. [1] (diamonds), Seynnes et al. [2] (plus), and Damas et al. [3] (circles). The chosen value of $k_M = 0.01\,\mathrm{h}^{-1}$ (green line) provides a good fit with the experimental data. Simulations were run on a cylindrical geometry with no feedback.

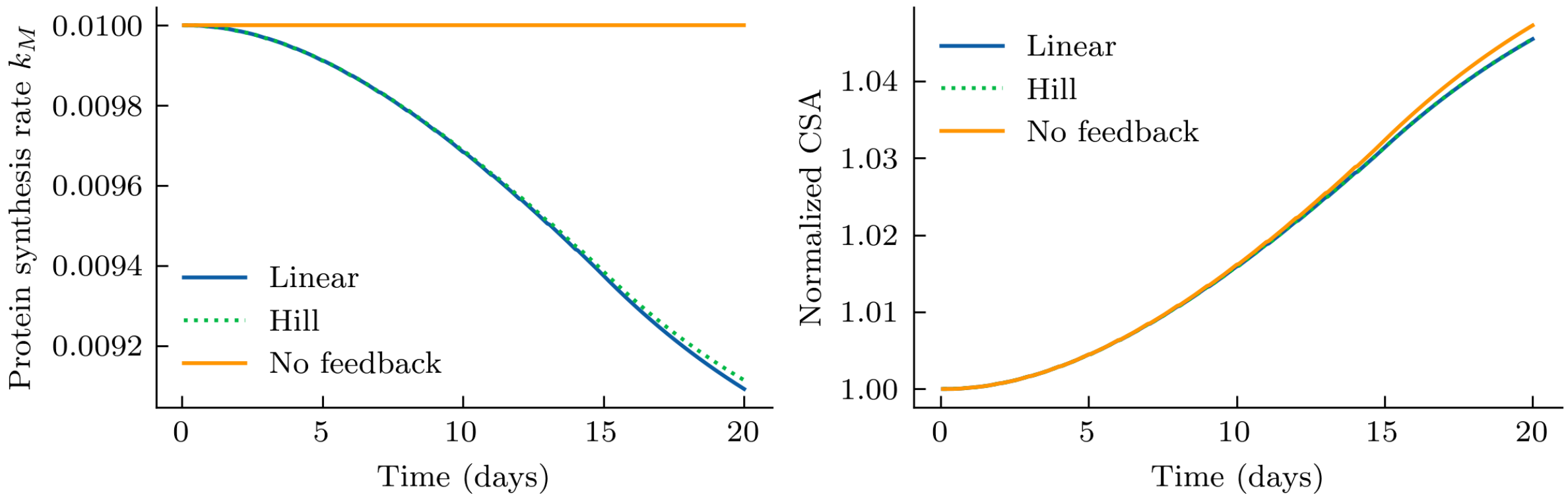

Fig D. Comparison of Hill-type, linear, and no feedback. The left panel shows the evolution of the protein synthesis rate, k_M, resulting from feedback based on the cross-sectional area, which is shown in the right panel. The simulations were performed on a cylindrical geometry using an exercise protocol of 1 hour of daily exercise for 15 days followed by 5 days of rest. Linear feedback was defined as $k_M(t) = (-2 \cdot A_{CS}(t)/A_{CS}(0) + 3)k_M^0$.

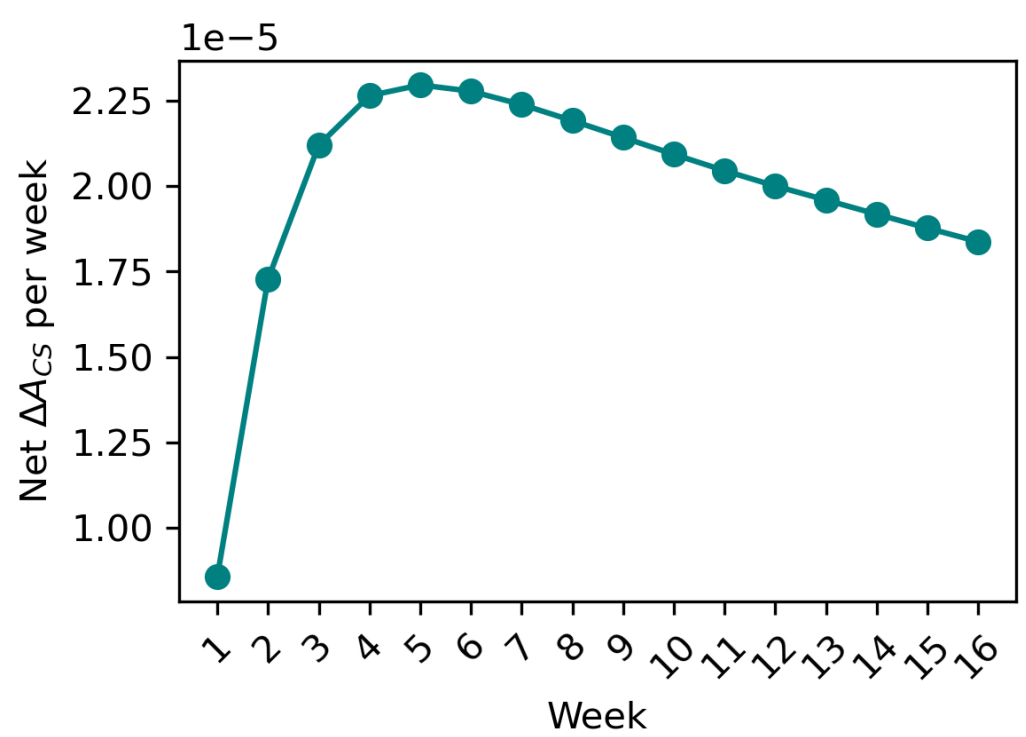

Fig E. Illustration of reduced growth rate due to feedback. The plot shows the weekly change in CSA for a 16-week MWF exercise protocol in the idealized fusiform geometry. The rate of growth decreases over time due to the feedback reducing the protein synthesis rate k_M as the cross-sectional area increases.

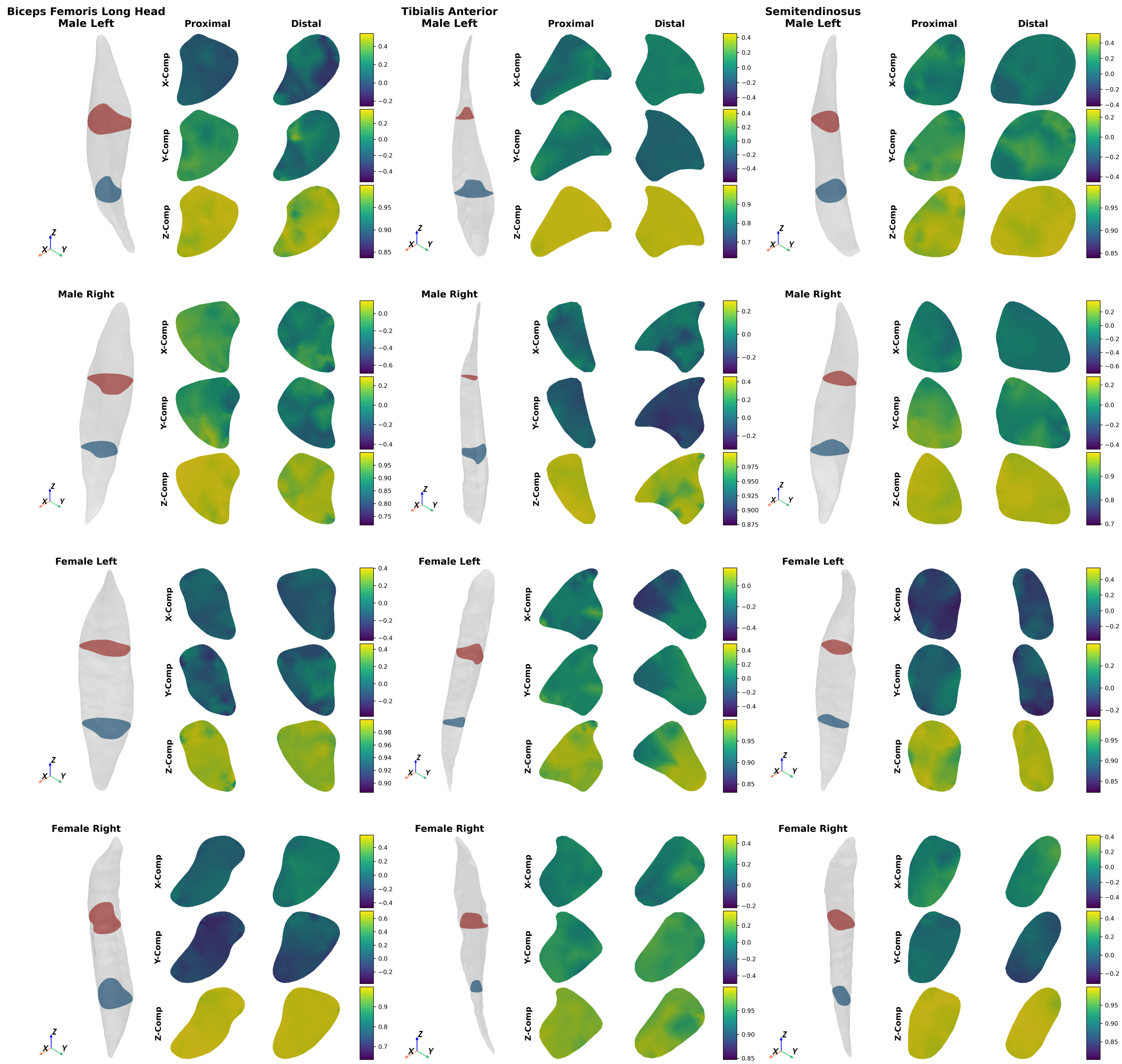

Fig F. Illustration of muscle fiber directions. Idealized fiber vector fields generated via Stokes flow for all 12 simulated geometries. Muscles are grouped by anatomical type (columns) and subject variation (rows). For each, a 3D outline shows the extraction of a proximal (red) and distal (blue) cross-section. Heatmaps display the transverse (X, Y) and longitudinal (Z) components of the normalized fiber vector. Color scales are globally bounded to the full respective 3D mesh.

Algorithm A. Cumulative growth implementation. Pseudocode for the cumulative growth simulation combining the signaling ODE model with the hyperelastic finite element model.

Initialize $\mathbf{G}_k, \mathbf{G}^{(k)}, \mathbf{A}^{(k)}, \mathbf{F}^{(k)}$ to identity tensors.
for (exercise, growth period) pair **do**
    Simulate ODEs for exercise and subsequent growth period.
    Divide growth period in steps of size Δt.
    for growth step k in growth period **do**
        Construct incremental growth tensor $\mathbf{G}_k$ from ODE solution.
        Update total growth tensor $\mathbf{G}^{(k)} = \left(\mathbf{A}^{(k-1)}\right)^{-1} \cdot \mathbf{G}_k \cdot \mathbf{A}^{(k-1)} \cdot \mathbf{G}^{(k-1)}$.
        Solve hyperelastic model for $\mathbf{F}^{(k)} = \mathbf{A}^{(k)}\mathbf{G}^{(k)}$.
        Update total elastic tensor $\mathbf{A}^{(k)} = \mathbf{F}^{(k)}(\mathbf{G}^{(k)})^{-1}$.
        Compute current cross-sectional area $A_{CS}(t)$.
        Update protein synthesis rate k_M through feedback function.
    end for
end for

References

1. DeFreitas JM, Beck TW, Stock MS, Dillon MA, Kasishke PR. An Examination of the Time Course of Training-Induced Skeletal Muscle Hypertrophy. European Journal of Applied Physiology. 2011;111(11):2785-90. doi:10.1007/s00421-011-1905-4.

2. Seynnes OR, De Boer M, Narici MV. Early Skeletal Muscle Hypertrophy and Architectural Changes in Response to High-Intensity Resistance Training. Journal of Applied Physiology. 2007;102(1):368-73. doi:10.1152/japplphysiol.00789.2006.

3. Damas F, Phillips SM, Lixandrão ME, Vechin FC, Libardi CA, Roschel H, et al. Early Resistance Training-Induced Increases in Muscle Cross-Sectional Area Are Concomitant with Edema-Induced Muscle Swelling. European Journal of Applied Physiology. 2016;116(1):49-56. doi:10.1007/s00421-015-3243-4.
